# Energy Transfer Pathways in Plant Photosystem I from First-Principles Modeling

**DOI:** 10.1021/jacsau.6c00568

**Published:** 2026-08-11

**Authors:** Elena Betti, Lorenzo Cupellini

**Affiliations:** Dipartimento di Chimica e Chimica Industriale, Università di Pisa, via G. Moruzzi 13, 56124 Pisa, Italy

**Keywords:** Photosystem I, PSI, red forms, excitation energy transfer, light harvesting, QM/MM, photosynthesis

## Abstract

The photosynthetic supercomplex Photosystem I–light harvesting complex I (PSI-LHCI) of plants is a molecular machine involved in energy conversion. Concentration of energy in the PSI reaction center is strikingly efficient, despite the unusual presence of low-energy, trapping states (red forms) spatially far from the reaction center. The main energy transfer processes occurring in PSI-LHCI are still unclear, as the size and complexity of the system prevent immediate interpretation of spectroscopic signals and challenges traditional modeling approaches. Here we present a multiscale quantum chemical model able to characterize the exciton structure of the full Chlorophyll aggregate in plant PSI-LHCI, including the red forms. The calculation of transfer rates between all pigments allows us to simulate population decay upon different initial conditions and to ultimately identify the most relevant, disorder-robust pathways at molecular resolution. The time scales associated with such pathways closely match the ones extracted from experiments, demonstrating the effectiveness of a quantum mechanical description. The inclusion of charge-transfer states in the red sites of the LHCI antennas allows us to characterize the role of red forms in the dynamics and quantify their effect on the overall efficiency.

## Graphical abstract

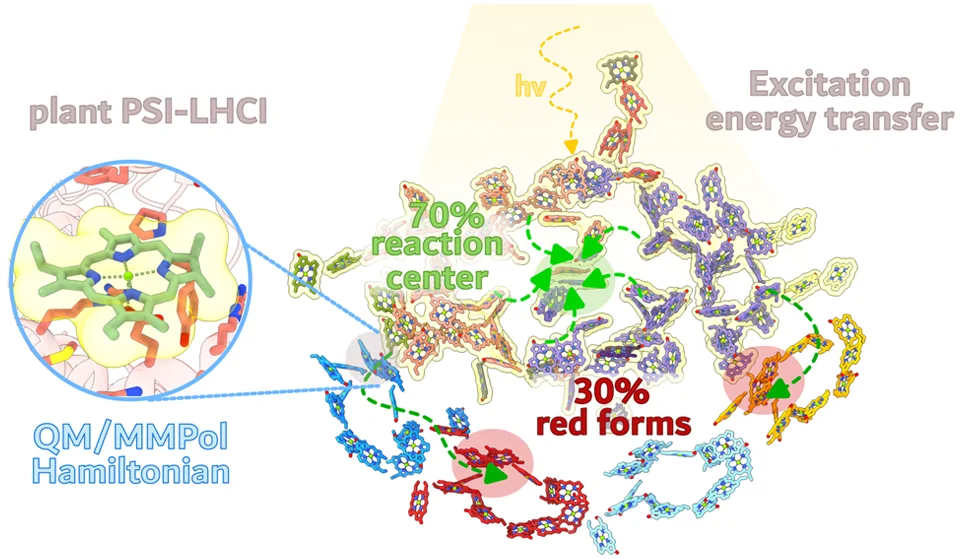

## 1 Introduction

Photosystem I (PSI) is a protein supercomplex found in the thylakoid membranes of algae, cyanobacteria and plants. It uses solar energy to perform the reduction of ferredoxin and the oxidation of plastocyanin.^2–4^ In plants and green algae, PSI comprises a monomeric core complex, which hosts the catalytic reaction center (RC) and coordinates about 100 chlorophyll (Chl) pigments, plus a belt of four light-harvesting complexes surrounding the core, which together make the LHCI antenna^5–8^ (see Figure 1A). LHCI is actually formed upon aggregation of two protein heterodimers from the Lhca family (Lhca1/4 and Lhca2/3), which coordinate in total about 50 more Chls, increasing the absorption cross section of the core.^9^ After light absorption by LHCI, the energy is quickly conveyed via excitation energy transfer (EET) toward the RC, where charge separation occurs.^4^ The light-harvesting efficiency (number of emitted electrons per absorbed photon) of PSI is among the highest in nature.^10^

**Figure 1:**
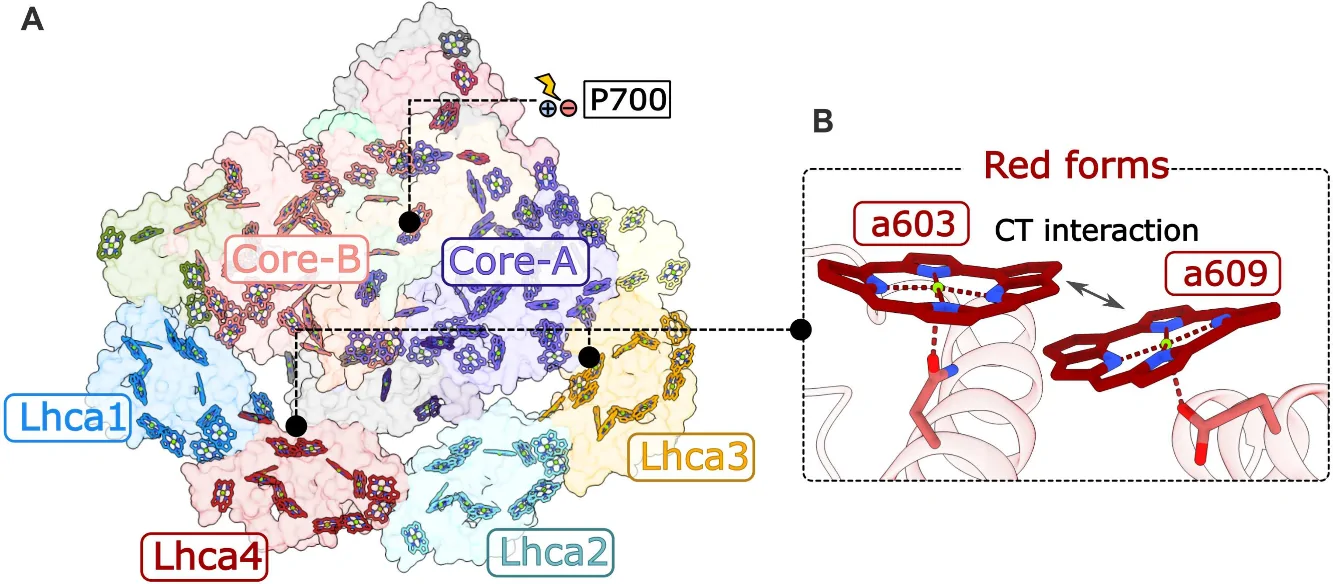
(A) Structure of the PSI supercomplex.^1^ The 156 Chl rings are shown together with the protein surfaces, colored by chain. The location of the P700 special pair is also indicated. (B) The a603-a609 Chl dimer in Lhca3 and Lhca4 responsible for the appearance of the red forms.

The remarkable efficiency of plant PSI looks rather counterintuitive when considering that the LHCI antenna features unusually red-shifted excited states, called red forms (RFs).^11^ These states are lower in energy than the special pair P700 responsible of the initial charge separation in the RC, and are thus expected to slow down the energy transfer to the RC. Spectroscopic^12,13^ and mutation experiments^14,15^ as well as quantum chemical calculations,^14,16,17^ have linked the RFs to the coupling of locally excited states with low-lying charge transfer (CT) states within a closely packed Chl dimer (Chls a603-a609) in the Lhca3 and Lhca4 antennas (see Figure 1B). Single-molecule spectroscopy experiments performed on Lhca3 and Lhca4 have revealed that the “red” conformation which presents RFs is in equilibrium with a “blue” one, lacking the RFs.^18^

The overall EET dynamics of plant PSI-LHCI supercomplexes has been investigated through time-resolved techniques, such as time-resolved fluorescence,^19–22^ transient absorption,^23,24^ and two-dimensional electronic spectroscopy (2DES).^21,25^ Analysis of the time-resolved data provided a consistent picture of the characteristic time scales of the EET dynamics: in the isolated PSI core, excitation trapping requires about 20 ps, while this time increases to 50 ps upon coordination of LHCI.^19,21,26,27^ Red forms in LHCI increase the trapping time, which is about double that expected only based on the extended antenna.^19,21,25^ These experiments have provided a clear but schematic understanding of the EET pathways in PSI-LHCI. In fact, there remain ambiguities in the interpretation of the data as a consequence of the system’s complexity. Since experiments give bulk information and microscopic parameters are not known, time-resolved data have to be interpreted with coarse-grained kinetic models which rely on multiple assumptions.^28^ Finally, the conformational (red/blue) inhomogeneity complicates the analysis by introducing additional uncertainty on the dynamics.

Computational models, both phenomenological^29,30^ and atomistic,^31–37^ have proven able to describe the photophysics of many photosynthetic proteins and therefore make powerful tools to disentangle the EET processes and link them to their spectral signatures. In PSI, earlier computational studies have focused on cyanobacterial PSI core complexes,^38–41^ for which high-resolution structures were available. Despite the good agreement with experimental spectra, these models yielded fairly different energetic orderings of Chls. Isolated Lhca4 of plants was studied with the main aim of understanding the origin and functional behavior of RFs.^17,42,43^ Recently, Novoderezhkin and Croce^44^ investigated the Lhca1-Lhca4 dimer and hypothesized preferential pathways from the dimer to the core complex. However, they obtain the Hamiltonian by fitting the experimental spectra of isolated antennas,^16,44^ allowing only a qualitative estimation of EET rates. Overall, a comprehensive computational model to investigate energy transfer in the entire plant PSI supercomplex is still missing.

Recently, we developed an atomistic, quantum chemical model of plant PSII supercomplex, which could reproduce experimental absorption and time-resolved fluorescence of the complex while providing molecular insights on the transfer dynamics.^45^ Here, we employ a similar approach to obtain a first-principles Hamiltonian for all the Chls in PSI-LHCI. This Hamiltonian reproduces the time scales of the EET events observed spectroscopically, and allows us to fully characterize the EET network of PSI.

## 2 Results and Discussion

### 2.1 Exciton Structure of PSI from First-Principles Atomistic Calculations

We first refined the X-ray structure of *Pisum sativum* published by Mazor et al.^1^ using the procedure developed in our previous work,^45^ which allows achieving a better description of the protein pockets hosting the pigments. Then, we employed state-of-the-art polarizable quantum mechanics/molecular mechanics (QM/MM) calculations to obtain the site energies corresponding to the Q_*y*_ transition of all Chls in the supercomplex and the excitonic couplings between them. The resulting site energies for the Chls aggregate are illustrated in Figure 2A (see also Figure S3 and Figure S4 of the SI), together with interpigment couplings larger than 15 cm^–1^.

**Figure 2:**
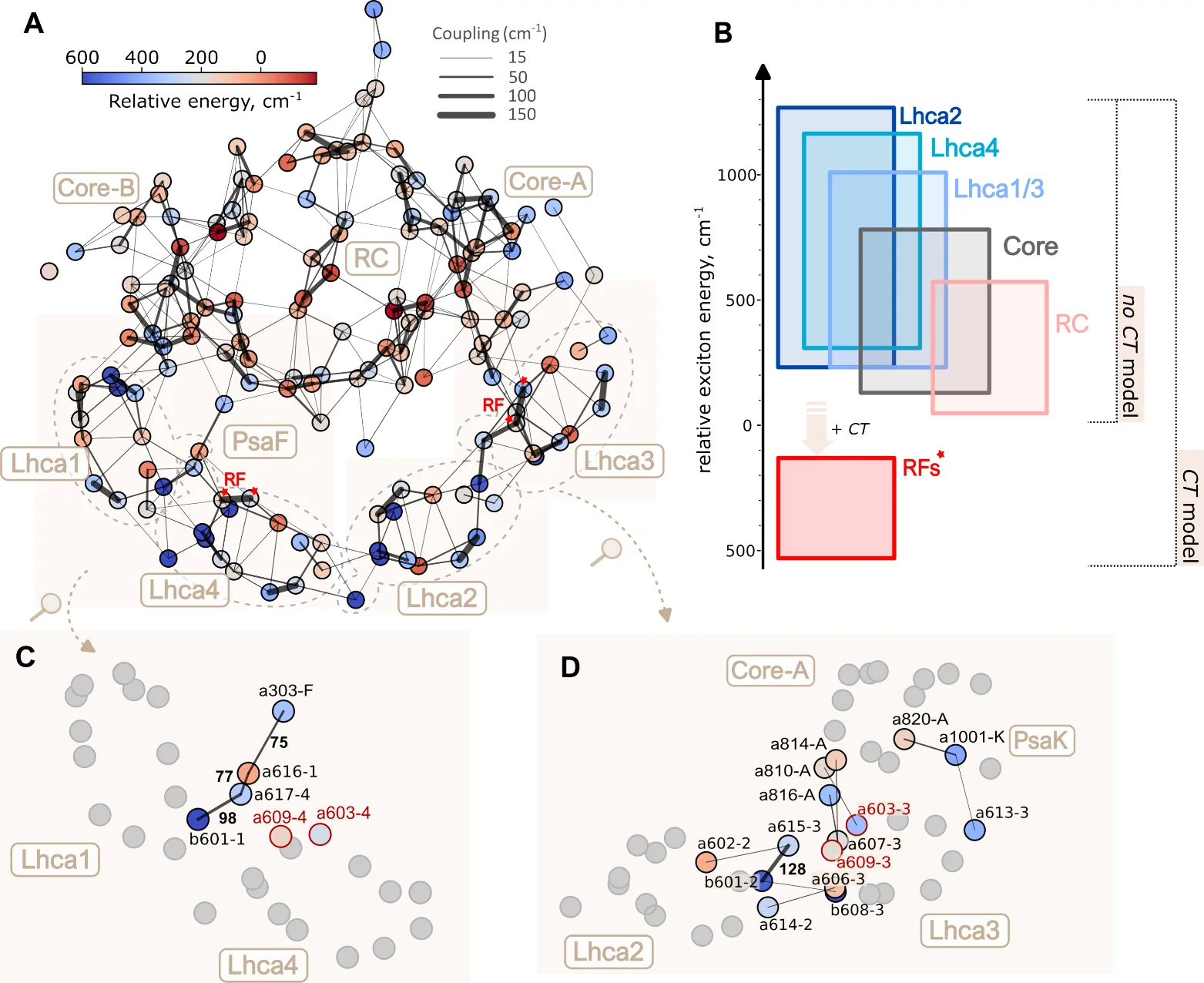
Exciton Hamiltonian of PSI supercomplex. (A) Chls shown as circles and colored according to their excitation energy relative to the average one of P700 pigments. Black lines represent couplings between the pigments they connect, with thickness proportional to the magnitude of the coupling. Only couplings larger than 15 cm ^–1^ are shown, and a scale is given on the upper right of the panel. (B) Overall exciton structure in the PSI supercomplex. Each box encompasses the distribution of all exciton states assigned to pigments within a given complex, broadened by static disorder (see Figure S1). The zero energy on the *y* axis is chosen to match the lowest state of the RC. (C, D) Zoom into some interprotein interfaces. Only interprotein couplings are shown, with the pigments participating in those couplings colored and labeled. For core Chls, we use the residue numbering found in the structure by Mazor et al.^1^ (PDB: 5L8R); for LHCI, we employ the numbering typical of Lhcb antennas.^5,46^ The red sites in Lhca3 and Lhca4 are also indicated with red labels for reference.

Our calculations predict that several pigments in the core (both chains A and B) have site energies close to those of P700 in the RC, some being even more red-shifted. In particular, Figure 2A shows a ring of low-energy Chls surrounding the RC from all sides. The pigments of this low-energy ring are only weakly coupled to the six Chls of the RC, and two Chls of higher energy lie between the ring and the RC, similar to cyanobacterial PSI cores.^38,41^ Various pigments of the core feature very large couplings ( > 100 cm^–1^), leading to the formation of extended domains of exciton delocalization.

Pigments in Lhca antennas have generally higher energy. This is especially true for Lhca4 and Lhca2, which have the highest content of Chls b. The largest couplings are found within single antennas, yet many non-negligible couplings provide efficient connections between Lhcas. The largest intercomplex couplings are within Lhca2/3 and Lhca1/4 heterodimers (see Figure 2C,D): a615-Lhca3/b601-Lhca2 (128 cm^–1^), b601-Lhca1/a617-Lhca4 (98 cm^–1^), and a617-Lhca4/a616-Lhca1 (77 cm^–1^). For the sake of clarity, we use for LHCI the Lhcb-based Chl numbering.^5,46^

We now analyze the couplings between the core and the LHCI antennas. Lhca1 and Lhca3 are well connected with the core through a network of couplings in the 20–40 cm^–1^ range, with the largest coupling (75 cm^–1^) found for a303-PsaF/a616-Lhca1 (Figure 2C). Lhca2 and Lhca4, at the center of the LHCI belt, do not show any relevant direct coupling to the core, but they are both connected to Lhca1 and Lhca3, as highlighted above. In particular, Lhca4 is coupled to Lhca1 through Chl a617, so that the a617-Lhca4 → a616-Lhca1 → a303-PsaF pathway makes a plausible candidate for Lhca4-core EET, as previously proposed.^44^

To analyze the excitonic structure of PSI, we consider the effect of energetic disorder, which yields a more realistic picture of exciton delocalization. To this end, we analyzed an ensemble of Hamiltonians obtained by allowing random shifts of the site energies, and analyze the exciton states in this disordered picture (see Methods). A comprehensive scheme of the exciton domains surviving disorder is shown in Figure S5 of the SI. In most cases, we find domains comprising 2 to 4 pigments. Within single Lhcas, Chls a603 and a609 and Chls a610, a611 and a612 often share large couplings and form low energy domains, as previously found.^17,44^ Despite the significant couplings, the Chl a/b pairs at the interface between Lhcas, a615-Lhca3/b601-Lhca2 and b601-Lhca1/a617-Lhca4 do not form exciton domains, given the large site energy difference. Conversely, the pair a617-Lhca4/a616-Lhca1 does form an interprotein exciton domain: our calculations thus reinforce the role of a617-Lhca4/a616-Lhca1 as a bridge from Lhca4 to Lhca1 and then to the core. This was not known *a priori*, since Chl a616-Lhca1 is likely lost during purification, and therefore its site energy (and consequently the exciton delocalization) could not be estimated from isolated Lhca1.^44^ We finally note a shared exciton domain between Lhca3 and the core, a613-Lhca3/a1001-PsaK (Figure 2D) which is expected to have smaller influence on the dynamics as it contains high-energy Chls.

A summary of the overall exciton energy landscape is shown in Figure 2B. Therein, the boxes account for the distribution of all exciton states assigned to pigments within a given complex (details in Section S2), broadened by static disorder. A funnel-like structure can be noticed, where the highest excitons belong to the antennas – mostly to Lhca2 and Lhca4 which contain most of the Chls b – and the lowest excitons are found in the RC. Note that while some Chls have lower site energy with respect to P700, the lowest *exciton* states actually belong to the RC. This reveals an important role of the couplings in determining the energy gradient in the core complex.

The Hamiltonian described so far only includes local excitations, and lacks the CT states responsible for the RFs. From now on, this model will be referred to as the *no CT* model. Arguably, the *no CT* model can describe well the blue conformations of Lhca3/4 likely present in experimental samples.^18^ In order to describe the red conformations, we extend the exciton Hamiltonian above with CT excitations within the a603-a609 dimer of Lhca3 and Lhca4 (from now on, *CT* model). The microscopic quantities needed to extend the Hamiltonian were taken from a previous work on Lhca4 ^17^ and assumed to hold also for Lhca3, given the high similarity between the two antennas.^19,47^ More details on the models are given in Section S2 of the SI.

The inclusion of the CT states in the Hamiltonian leaves unaltered the electronic structure of the aggregate, except for the appearance of the RFs, low-energy excitons with mixed CT character (see Figure 2B). Their distribution is broader than that of other complexes, considering the small number of pigments in the box, due to the larger coupling of CT states to the environment.^17^ The *CT* and *no CT* models provide us with two limiting cases that are present in realistic samples, although in practice we do not know their relative weight. Nevertheless, comparison of the two models allows us to directly quantify the effect of the RFs on the optical and dynamical properties of PSI.

### 2.2 Optical Spectra and Decay Dynamics

In Figure 3A we show the experimental absorption spectrum of the PSI supercomplex,^48^ together with the one simulated from the two model Hamiltonians, *CT* and *no CT*, and averaged over disorder. The transparent curves superimposed on the spectra quantify the contribution to the total spectrum of the sum of pigments a603 and a609 in Lhca3 and Lhca4, for the *CT* (violet) and *no CT* models (slate blue). A comparison between simulated and experimental spectra on isolated Lhcas and core complex can be found in the SI (Figure S6). The *CT* and *no CT* models are nearly identical at wavelengths below 700 nm, and appear in very good agreement with the experiment. At longer wavelengths, the *CT* model shows a large band extending above 720 nm, which arises from absorption of the RFs, as shown by the contributions to the spectra. Upon including CT states in the Hamiltonian, excitons on a603-a609 are red-shifted by about 20 nm. When comparing the two models with the experiment, it becomes clear that the inclusion of CT states in the Hamiltonian is crucial to reproduce the red region of the experimental spectrum. Therefore, we assign this absorption to the two RFs.

**Figure 3:**
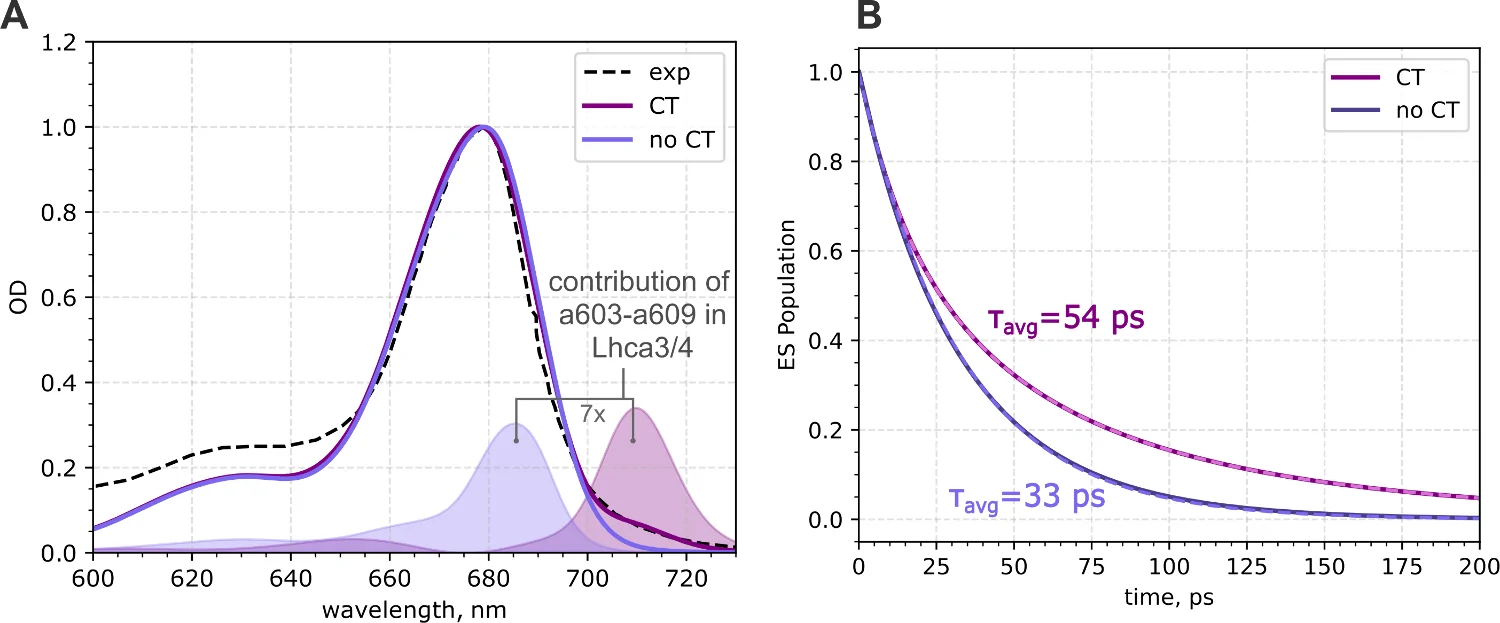
Photophysical properties of PSI supercomplex. (A) 290 K absorption spectra of the supercomplex simulated with the *CT* and *no CT* models, shown along with with the experimental spectrum^48^ (dashed). The contribution to the total spectrum by Chl a603 and a609 from Lhca3 and Lhca4 in the *CT* and *no CT* models is shown as a shaded area (scaled by a factor of 7). All simulated spectra were rigidly shifted by −1540 cm^–1^ to account for the systematic error of the QM method. (B) Room temperature decay of the excited state population simulated with the *CT* and *no CT* model (solid) and exponential fitting (dashed).

In order to study the EET dynamics in the two conformations, we consider two kinetic models of the PSI-LHCI supercomplex, obtained from either the *CT* or the *no CT* model Hamiltonian. For each realization of the disorder in *CT*/*no CT* Hamiltonians, we compute rates between all states using the Redfield-Förster method, which combines Redfield and Förster EET theories for transfers within and between strongly coupled pigment domains (details in Section S4). The domains are those found upon analysis of the exciton Hamiltonian and shown in Figure S5 of the SI. Given the complexity and multistate nature of the charge separation process in the RC,^49^ we describe it phenomenologically and add to the kinetic models a trap state, connected to the lowest exciton of the RC domain with a fixed rate of 2 ps^–1^. This value was chosen as it results in a 1 ps^–1^ rate from the RC *domain* to the trap, which was estimated as the effective charge separation rate at room temperature in a recent spectroscopic analysis^50^ (more detail in Sections S4.1 and S4.3 of the SI).

In Figure 3B we show the decay of the total excited state population (excluding the trap state) simulated at room temperature after homogeneous excitation and averaged over the disorder. The decay time corresponds to the trapping time, namely the average time needed for an initial excitation to be trapped in the RC. As expected, the *CT* model yields a slower decay with respect to the *no CT* case, due to the low-energy RFs. A multiexponential fitting of the two curves yielded an average time of 33 and 54 ps for the *no CT* and *CT* models, respectively. Time resolved fluorescence spectroscopy measurements revealed an average fluorescence decay time around 48–55 ps for samples of PSI supercomplexes.^19,21^ This well compares to our time scales obtained above, in particular if we assume a prevalence of the red conformation in the experimental sample. Importantly, the very good agreement between simulations and experiments for both spectra and time scales validates our computational model for analyzing the EET dynamics and pathways.

In the following, we compute and compare trapping times in the *CT* and *no CT* models (τ_CT_ and τ_noCT_) starting from different excitation conditions at room temperature. An analysis of the temperature dependence of these results can be found in Section S6 of the SI.

The results are shown in the first two columns of Table 1; the difference (Δτ) and ratio between the *CT* and *no CT* (the slowdown factor), are also reported to ease the comparison. Migration times from each Chl pigment are shown in Figure S8 of the SI.

**Table 1:** Trapping Times (in ps) within the *CT* and *no CT* Models and Fraction of Excitation Visiting the RFs within the CT Model, upon Specified Initial Conditions

Initial excitation	τ_CT_	τ_noCT_	Δτ	τ_CT_/τ_noCT_	f_RF_
Homogeneous	54	33	22	1.6	41%
LHCI blue	81	42	39	1.9	70%
a603/a609 Lhca4	123	45	78	2.7	94%
a603/a609 Lhca3	71	38	33	1.9	96%
Core	41	28	13	1.5	29%

In addition to the homogeneous excitation discussed above, we consider the initial excitation of all pigments in LHCI antenna, excluding those involved in the RFs (hereafter, “LHCI blue”). In both models, the trapping time is longer than for the homogeneous excitation. In addition, here the slowdown of the dynamics upon inclusion of the RFs is more evident, as the slowdown factor increases from 1.6 to 1.9. An initial excitation on the core reaches the trap faster than an initial homogeneous excitation, with a trapping time of 41(28) ps in the *CT*(*no CT*) model. The difference in trapping time between core and antenna excitation (14–40 ps) compares reasonably well with the same difference extracted from time-resolved fluorescence (∼20 ps).^19^ Notably, the trapping time from the core increases by almost 50% when CT states are included. This suggests that at least part of the excitation created in the core leaks to the LHCI antenna and visits the RFs before returning in the core and being trapped in the RC, increasing the trapping time. This rather counterintuitive back-flow of excitation from the core to the antennas was also observed in PSII supercomplexes.^45,51^

Direct excitation of the a603-a609 dimer in Lhca4 yields the largest slowdown factor of 2.7. This factor agrees well with the 3-fold increase of the average trapping time measured in 2DES experiments when the RFs are preferentially excited (around 715 nm).^25^ Interestingly, our simulations show a different behavior of Lhca3 and Lhca4 red forms. Directly exciting the Lhca3 red Chls results in a smaller slowdown factor (1.9 instead of 2.7) and Δτ (33 ps against 78 ps). If we interpret the latter as a measure of the “effective time” required for an excitation to escape from the RFs, we can conclude that RFs in Lhca4 make much more effective traps for the excitation than those in Lhca3. As the CT parameters in our model are identical for Lhca3 and Lhca4, the observed difference must arise from the topology of the EET network, that is, the better connectivity of Lhca3 with the rest of the supercomplex.

To explain the increase in trapping time due to the RFs, we report in Table 1 the fraction of excited-state population reaching the RFs (*f*_RF_) before the trap, upon each different starting condition (see Section S4 for details). Large part (∼70%) of the excited-state population created in LHCI visits the RFs before being trapped, while the rest is directly conveyed to the core. This indicates that efficient antenna-core connections exist, and shows that the excitation does not equilibrate within LHCI before transferring to the core. Upon excitation of the core instead, ∼ 30% of the initial excitation ends up in the RFs before reaching the RC trap, confirming that excited-state population can flow back from the core to the antennas. Notably, a homogeneous excitation of the a603-a609 pigments still allows a small fraction of the population to escape the RF (Table 1) by direct energy transfer to neighbors.

For completeness, we note here that our kinetic model only includes Chl pigments and therefore neglects EET pathways involving carotenoid pigments. While these latter can mediate indirect Chl to Chl transfer as well as dissipate excitation by rapid internal conversion to the ground state, these processes are expected to play a secondary role compared to the main Chl to Chl EET routes and trapping in the RC.

### 2.3 Energy Transfer Pathways toward the Reaction Center

The results discussed so far prompt us to better investigate the connections between antennas and core and within LHCI. A molecular-scale understanding of the EET pathways can be gained through the calculation of population fluxes, which indicate the main pathways followed by the excitation in reaching the RC trap.^45^

Briefly, the flux Φ_*ab*_ from exciton *b* to exciton *a* quantifies the net expected number of hops from state *b* to state *a* that occur throughout the dynamics. For example, a flux Φ_*ab*_ = 1/2 means that half of the excited-state population passes from the *b* → *a* channel. In order to compute averages over the disordered ensemble of Hamiltonians, in which the excitons change realization by realization, we then compute fluxes between Redfield-Förster exciton domains and average the latter over the ensemble (the mathematical definition of fluxes can be found in Section S4). We note that fluxes defined in this way only quantify net movements of excitation: if, for example, the excitation moves back and forth along the same channel, the associated overall flux is zero.

In Figure 4 we show as weighted arrows the net flux between pigment domains from different excitation conditions. These indicate the pathways most likely followed by an exciton starting from each initial condition. Upon initial excitation of the core (Figure 4A), the energy reaches the reaction center in a “bike-wheel” fashion from the surrounding low-energy Chl ring discussed above. This scheme is at odds with what observed e.g. in plant Photosystem II, where an essentially linear pathway conveys the excitation from the periphery to the RCs.^45,52,53^ The backflow process mentioned above is not directly visible in this representation, as the ∼ 30% population that visits the RFs travels back to the core before being eventually trapped in the RC, resulting in a null net flux.

**Figure 4:**
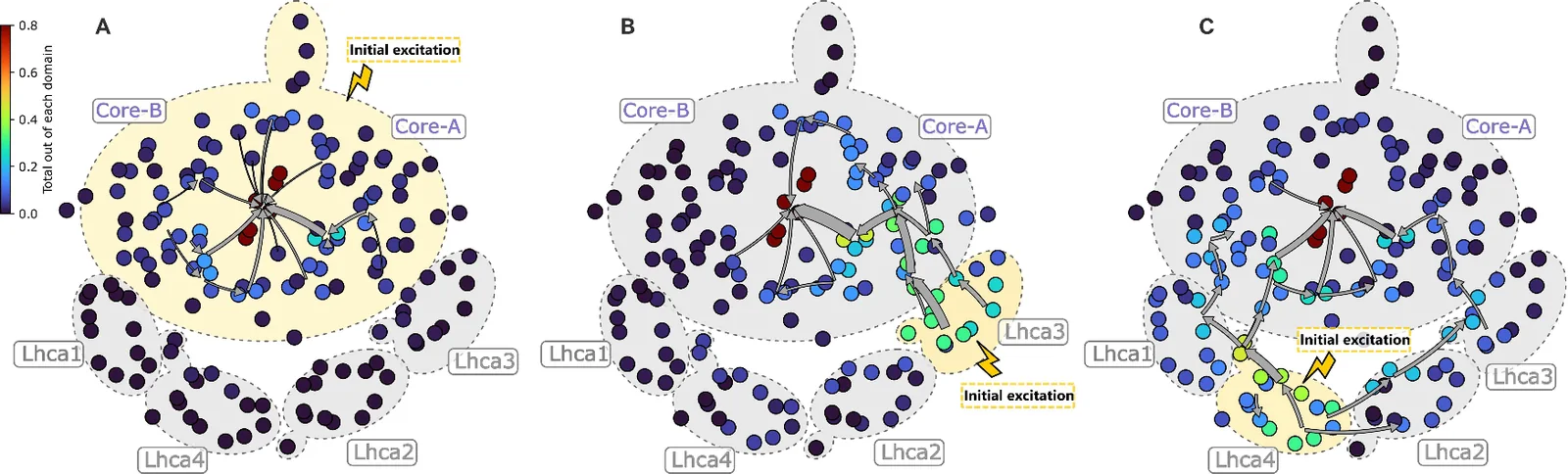
Population flux upon different starting conditions. (A) core excitation, (B) Lhca3 excitation, and (C) Lhca4 excitation. In all cases, the arrow width is proportional to the flux, and the color of each circle indicates the total flux out of the domain (see the color bar). Only fluxes above 3% in (A) and 7% in (B) and (C) are shown.

The excitation of different Lhcas in LHCI results in different pathways to the RC, as a consequence of the different connectivity of each Lhca with the core. Initial excitation of Lhca3 (Figure 4B) results in one primary channel from the antenna to the closest chain of the core (chain A), with only minor transfers crossing the LHCI belt. A similar picture is obtained exciting Lhca1, this time with the primary channel passing through Core-B (see Figure S7). Conversely, the excitation of Lhca4 (Figure 4C) results in a branching of the population, which reaches the RC via Lhca1 → Core chain B, or via Lhca2 → Lhca3 → Core chain A. No significant direct flux is visible from Lhca4 to the core, consistent with the absence of sizable couplings (Figure 2A). However, an important part of the excitation is conveyed to the domain shared with Lhca1 (a616-Lhca1/a617-Lhca4), from which the core is reached through either a303-PsaF or another low energy domain (a602-a603) in Lhca1. Initial excitation of Lhca2 (see Figure S7) yields a branching across LHCI similar to that upon excitation of Lhca4. Lhca2 also features a very small direct connection with the core through bridging Chls in PsaJ and PsaF.

It is reasonable to believe that strong connections with the core allow dynamical population and depopulation of the Lhca3 RFs (at room temperature), whereas the more isolated position of Lhca4 results in RFs behaving more like a disconnected energy basin. This picture also explains the observation that PSI-Lhca1/4 supercomplexes lacking Lhca2/3 feature the same trapping time of ∼ 50 ps as PSI-LHCI:^19^ in fact, the addition of another red site upon binding of Lhca3 in PSI-LHCI is compensated by its optimal connections to the core, so that the slowest process is still associated with depopulation of RFs in Lhca4.

Upon homogeneous excitation of the antenna, a combination of the pathways found above occurs. To assess the asymmetry of plant PSI-LHCI, we computed population fluxes upon homogeneous excitation of the LHCI antennas, and obtained the fraction of the total population flowing to the core from each of the antennas. Interestingly, our model predicts that 45% of the initial excitation reaches the core from Lhca3, and 47% from Lhca1, highlighting an almost perfectly symmetric scheme of energy flow from the Lhca belt to the core. The remaining fraction reaches the core following minor pathways directly from Lhca2 (5%) and Lhca4 (3%).

### 2.4 Analysis of Energy Transfer Kinetics Reveals the Different Role of Lhca3 and Lhca4 Red Forms

In the previous analyses, we have tracked down the most probable EET pathways at molecular scale, by exploiting the atomistic nature of our model. Time-resolved experiments cannot distinguish between individual EET routes and in general provide time scales for population rise and decay. To interpret the experimental time scales in terms of molecular pathways, we extract from our kinetic model the main time scales associated with EET processes.

We focus on the dynamics occurring within the CT model, as it most likely represents the majority of the population in experimental samples and contains richer EET dynamics. The core idea is to analyze the kinetics within a simplified model, which considers the population of the following five compartments: RC domain, RFs of Lhca3 (comprising a603, a609, the CT states, and a615 which forms excitons with the red sites), RFs of Lhca4 (comprising a603, a609, the CT states, and a602), LHCI blue (all pigments in the antenna excluding the RFs) and the Core (the rest of the pigments). We fit the population decay of all compartments simultaneously, with the same exponentials exp­(−*t*/τ_*i*_). The associated amplitudes *A*_*i*_ allow assigning decay (negative sign) and increase (positive sign) of the population of each species in the time scale τ_*i*_,^34^ similarly to the global analysis of time-resolved spectroscopy.^28^ More details are given in Section S5 of the SI.

To disentangle different time scales, we consider an initial excitation of either core or LHCI antenna (Figure 5A,C). In Figure 5B,D we depict a scheme of the EET processes as emerged from our analyses. The EET times shown next to the arrows from the initially excited compartments to the directly connected compartments were estimated as a weighted average of the components showing rise of the population (positive amplitude) concomitant to the decay of the excited domain (data in Figure S10). The dashed lines from the RFs to the RC represent the total decay time, obtained by averaging the time scales with negative amplitudes. We stress the fact that such time constants do not correspond to genuine transfer rates among compartments, as a compartmental kinetic model would not reproduce the population dynamics.^45^ Instead, the time constants reveal the general transfer time scale observed with the chosen initial condition.

**Figure 5:**
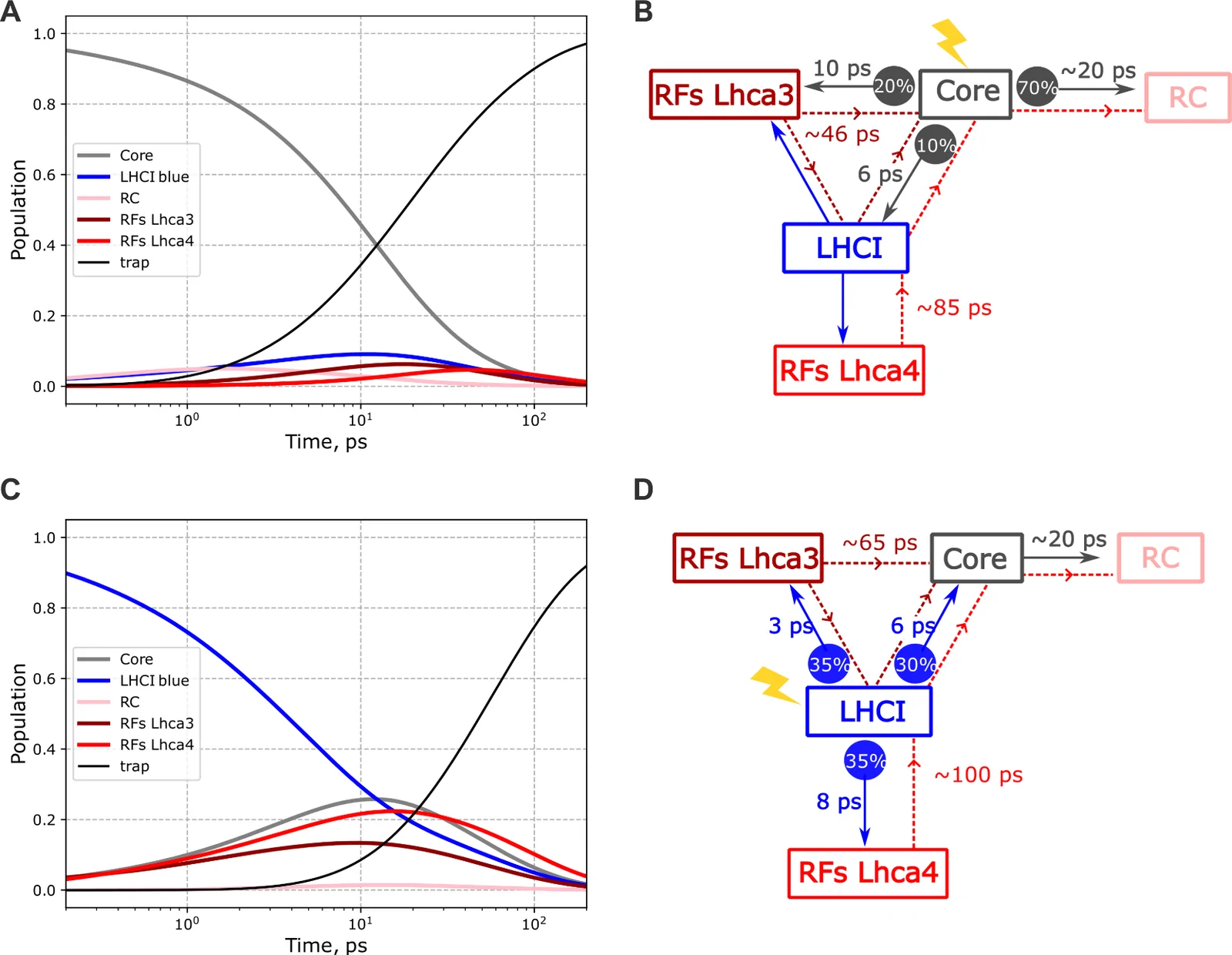
Analysis of population dynamics for core and antenna initial excitation. (A, C) Calculated population dynamics of the main compartments for (A) core excitation and (C) LHCI excitation (excluding the red forms). The population of the trap is also reported. Note the log-scale in the time axis. (B, D) Corresponding scheme showing the main EET events after excitation. The approximate time scale of the EET steps is shown beside the arrows. Dashed arrows represent the flow toward the RC of the population that has reached the RFs.

After excitation of the core, the population decays already within a few ps, populating LHCI and the RC (Figure 5A). The majority (70%) of the excitation decays in ∼20 ps by direct trapping in the RC (Table 1). This time constant was obtained from a model containing only the core (see Figure S11). The remaining fraction of excitation back-flows to the antennas and visits the RFs, which are both populated later in the dynamics (Figure 5A). 20% of the population visits the Lhca3 RFs, finally decaying within 46 ps, while the remaining 10% flows through LHCI and ends up in the Lhca4-RFs. This part of the population is eventually trapped back in the RC after ∼ 85 ps. It is clear from Figure 5A that RFs of Lhca4 are populated later than those in Lhca3, as the excitation needs to move through Lhca1 or Lhca2. They also depopulate more slowly, with an effective decay time which is roughly the double of that of Lhca3 RFs.

Upon excitation of LHCI (Figure 5C,D) both the core and RF populations rise quickly. The core is populated faster, but only 30% of the excitation transfers to the core without visiting the RFs. The remaining 70% transfers to the RFs in less than 10 ps. In this case the two RFs are visited roughly equally, although Lhca3 RFs are populated slightly faster than Lhca4 RFs (Figure 5C,D). Also in this case, Lhca4 RFs decay more slowly, approximately in 100 ps. Overall, the LHCI-core connections allow very fast transfers between antennas and core, at least for part of the excitation. This allows for an initial excitation on LHCI to avoid the RFs, and for an initial excitation on the core to reach the RFs. If the excitation reaches the RFs, though, its trapping time increases to 50–100 ps.

The analysis above roughly assigns an effective time to the pathways identified through the population fluxes. However, time-resolved experiments generally work under initial homogeneous (or nearly homogeneous) excitation.^19,21^ For such a homogeneous initial condition, we recovered six kinetic components with characteristic times of 0.6 ps, 3.7 ps, 13 ps, 32 ps, 81 and 258 ps (the amplitudes are shown in Figure S9 of the SI), which we use to interpret the experimental dynamics.

The shortest component (0.6 ps) explains the dynamics of a minor part of the total excitation and is dominated by the decay of population in LHCI in favor of the core and the RFs in both Lhca3 and Lhca4. Thus, this subpicosecond process can be interpreted as an ultrafast equilibration between well connected “blue” Chls in LHCI and bulk/red states in both core and antennas, and closely matches a 0.5 ps component found in 2DES^21^ and transient absorption^23^ of PSI-LHCI at room temperature, which was associated with equilibration of states within the main Chl a band. The 3.7 ps component mainly represents transfer from LHCI to the RFs. A similar time scale of 3–5 ps was also detected in the analysis of streak camera^19^ and 2DES^21^ measurement and associated with the decay of the bulk Chls and rise of species absorbing below 700 nm. A minor process in this time scale is an early Core → RC → trap transfer, which was also observed at this time scale in 2DES.^21^ The 13 ps component describes the main trapping phase from the Core, along with some transfer to RFs of Lhca3 mainly. This component can be associated with the 15–20 ps one found in experiments,^19,21^ although it is slightly shorter in our analysis.

The 32 ps component describes a decay of all compartments, except for the Lhca4 RFs which gains population. While in this time scale Lhca3 RFs decay, the Lhca4 RFs still gain population from other compartments. Only after ≈ 80 ps, the main decay from Lhca4 RFs does start. The 80 and 258 ps components represent the decay of residual population to the trap. According to time-resolved fluorescence measurements,^19^ RFs decay mainly in 80 ps, with an additional component around 230 ps corresponding to the emission maximum of Lhca4.

This analysis shows that our population dynamics does develop on the same time scales as found in experiments, and validates the pathways characterized above.

### 2.5 Discussion

Our first-principles atomistic model recapitulates the main spectroscopic and dynamic features of PSI-LHCI.^19,21–23^ In addition to the overall decay time, multiple time scales of different EET events are reproduced reasonably well. Remarkably, this is achieved from first-principles without introducing experimental biases or inputs from single antenna complexes. This approach allows us to model Chls at the interface, which are usually missing in single complexes, and to account for the effect of aggregation of the single complexes into PSI-LHCI. The model can provide a satisfactory description of the Q_*y*_ band only when it includes CT states in the a603-a609 dimers of Lhca3 and Lhca4, which give rise to the red forms. According to our assignment of spectral features and time components to RFs, we speculate that the “red” conformation is the dominant one at room temperature, while the “blue” one is minor. Conversely, in isolated Lhca4 monomers or Lhca1/4 dimers both conformations are significantly populated.^18^ Therefore, it seems likely that aggregation to PSI changes the conformational freedom of Lhca proteins.

The light-harvesting efficiency in PSI depends on the connectivity between antennas and the core, which, according to our simulations, is efficient for Lhca1 and Lhca3, allowing for fast EET from LHCI to the core and excitation backflow from the core to LHCI. We stress here that this backflow represents a nontrivial process for EET dynamics in PSI-LHCI. Indeed, our calculations reveal that full equilibration of the system does not occur before trapping in the RC, as demonstrated by the excitation-dependence of the trapping time (see Figure S8) and also supported by spectroscopic measurements.^54^ Hence, the backflow of excitation from the core to the antennas is not a consequence of excitation equilibration over the entire PSI-LHCI, and instead represents a novel mechanistic observation.

Lhca2 and Lhca4, which are not (or weakly) directly connected to the core, transfer their energy mostly to Lhca1 and Lhca3. Analysis of population fluxes further revealed the importance of the shared Lhca1/Lhca4 Chl a domain in the transfer toward the core. These differences in connectivity of LHCI antennas with the core were neglected in early coarse-grained modeling of PSI-LHCI supercomplexes,^19,21^ yet they explain the difference observed above in the trapping times upon excitation of Lhca3 and Lhca4 RFs.

Once in the core, efficient EET to the RC relies on a bike-wheel scheme to convey the excitation from all directions, by using as gates the ring of pigments surrounding the RC domain (See Figure 4). This design principle, very different from that of PSII, may explain the remarkable efficiency of PSI light harvesting.

Adolphs et al.^38^ predicted an asymmetric light harvesting process in the core of cyanobacteria, with a preference for energy reaching the RC from the A branch rather then the B branch. On the contrary, in our simulations, the excitation reaches the RC from both sides equally, with Lhca3 and Lhca1 functioning as symmetric gates from the LHCI antennas to the core chains A and B. While most of the protein sequence in the PSI core is conserved across cyanobacteria and plants, several point mutations can be observed in close proximity of the pigments (see Figure S12): given the high sensitivity of site energies to the electrostatic surrounding, we argue that those differences can easily result in a different energetic landscape in the two organisms.

Recently, Reiter et al.^41^ performed accurate QM/MM calculations of site energies of Chls in the cyanobacterial trimeric core, and observed a large variability in site energies across the frames. They concluded that the energetic landscape of PSI does not resemble a rigid funnel but rather a dynamic surface changing in the short time scale. Although EET rates were not directly computed, they predicted that the transfer to the RC occurs through pathways that are transiently formed and disrupted along site-energy fluctuations.

Here, we introduced a careful separation between fast and slow energy fluctuations.^55^ We treated fast fluctuations within an open quantum systems formalism, which allows us to account for exciton-vibrational coupling and detailed balance.^55–57^ Slow fluctuations, often referred to as static disorder, are instead accounted for by repeating all of our calculations on an ensemble of Hamiltonians sampled from a Gaussian distribution, assuming uncorrelated disorder on different sites.

To assess how the disorder affects the EET pathways, we compare in Figure S13–S14 the average population fluxes with those of single disorder realizations. For initial excitation on either Lhca3 or Lhca4, most of the EET pathways identified in Figure 4 can be still recognized in the individual realizations. However, their weight depends on the specific disorder realization. This demonstrates that the most likely pathways are rather robust over disorder, although their contribution can change. In other words, the pathways we identified do not vary dramatically from one realization to the other, contrasting the hypothesis of dynamically changing EET pathways. The fluctuations in the energy landscape are such that, at least at room temperature, the same pathways are almost always viable and preferred.

Our calculations revealed that red forms significantly influence the overall EET dynamics. Upon homogeneous excitation, the trapping time increases by ∼ 60% when CT states in Lhca3/4 are considered. This effect is larger the more the initial excitation can visit the RFs (see Table 1), in line with previous work.^25^ As the core is well connected to LHCI, an excitation created on the core can still end up in the RFs, substantially increasing the migration time to the trap. Our analyses suggest that RFs in Lhca4 slow down the trapping time more than Lhca3 RFs, even though in our simulations we have used the same CT parameters for both. This behavior can be explained with the larger connectivity of Lhca3 to the core. Notably, in an early spectroscopic study of PSI particles, Slavov et al. already distinguished the origin of two observed slow kinetic components by assigning them to RFs of Lhca3 and Lhca4;^26^ more recently, two “red” states with substantially different transfer rates to the core were proposed from the combined analysis of time-resolved fluorescence and transient absorption data.^23^ In any case, the RFs do not compromise the efficiency of the overall light-harvesting process: the trapping time in the CT model is still more than one order of magnitude smaller than the natural lifetime of Chls in the thylakoid membrane.^25,58,59^ In this way, the RFs can extend to the red the absorption window of the PSI antenna to the red side of the spectrum without significantly reducing the overall light-harvesting efficiency, as it was shown to occur in cyanobacteria.^60^ The role of plant RFs as red-light harvesters is also supported by evolutionary considerations: for example, organisms inhabiting environmental niches depleted in red light (e.g., the marine one), or simply less light-competitive, lack RFs.^61,62^ The different behavior found here for Lhca3 and Lhca4, however, suggests a different function for the two red sites, which merits further investigation.

By analyzing our population dynamics, we found the same time scales that were observed in spectroscopic experiments.^19,21–23^ Our analysis confirms the overall assignment of these kinetic components, but shows that the subps transfers only involve the most closely connected pigments of LHCI and core. This is confirmed by the shorter migration times found only for some LHCI chlorophylls (See Figure S8 in the SI). The overall equilibration of excitation within LHCI is slower than the transfers to the core or to the RFs, which allows part of the population to “escape” the RFs. Overall, these results indicate that EET in PSI-LHCI cannot be described as a hopping between equilibrated compartments. Compartmental models (Figure 5) can help in understanding the time scales observed spectroscopically to different EET processes. Nonetheless, the EET times extracted from these models or from experiments should not be interpreted as kinetic rate constants.

## 3 Conclusions

Our first-principles atomistic model of plant PSI-LHCI, based on accurate QM/MMPol calculations, provides a state-of-the-art picture of the energy landscape of the PSI-LHCI supercomplex, which reproduces spectra and dynamics. Notably, the agreement is not enforced by any fitting to experimental data, with the only empirical parameters being a global rigid shift of the spectrum and the effective trapping rate in the RC. Our results demonstrate that including CT states in the antennas is necessary to reproduce the red region ( > 700 nm) of the experimental absorption spectrum. The resulting red forms slow down the dynamics, and increase the trapping time even for an excitation started in the core. Our first-principles description further revealed the connectivity within the LHCI antenna and with the core: while Lhca1 and Lhca3 feature direct connections with the core, Lhca2 and Lhca4 exploit coupling with other Lhcas to convey the excitation to the core. This connectivity allows an excitation in the core to flow back to the antennas, similarly to what observed in photosystem II. Overall, our work aligns to what previously inferred from spectroscopic investigation, while providing a molecular-scale insight into the energy transfer dynamics of PSI.

## 4 Methods

### 4.1 Structure Refinement and Exciton Hamiltonian

All calculations were performed on a refined version of the structure of *Pisum sativum* PSI (PDB: 5L8R),^1^ which includes 156 Chl pigments. The structure refinement, based on a combination of restrained force field-based minimizations and simulated annealing cycles, has been already described in a previous work by the authors.^45^ Briefly, this refinement allows relaxing some parts of the crystal structure (e.g., protein side chains in the pigment pockets) to a more physical conformation.

As explained above, we explicitly modeled the blue and red conformations with a different exciton Hamiltonian. For the *no CT* model, the full exciton Hamiltonian of the 156 Chl pigments was modeled through a standard Frenkel exciton model. Q_*y*_ excitation energies and couplings were computed using a QM/MMPol approach^63,64^ implemented in a locally modified version of Gaussian 16.^65^ The QM region including the Chl ring and treated at the TDDFT (M06-2X^66^) level. The surrounding environment up to 30 Å from the ring was explicitly considered, and a cutoff of 15 Å from the pigment was considered polarizable. To model the *CT* conformation, we extended the Hamiltonian to include CT states formed within the Chl dimer a603-a609 in the Lhca3 and Lhca4 antennas. This implies the addition of 4 new states, 2 for each dimer to account for the two possible polarities of the states. These states are only coupled to the Q_*y*_ states of the pigments participating to the dimer. Their energy and the magnitude of the couplings were taken from a previous work^17^ (details in Section S2). To reproduce inhomogeneous (or static) disorder, we created an ensemble of 500 Hamiltonians (one for the *CT* and one for the *no CT* model) by assuming independent random shifts for all site energies sampled from a normal distribution centered in 0 and with $\sigma_{Q_{y}}=$ 100 cm^–1^ for Q_*y*_ states and σ_*CT*_ = 600 cm^–1^. These values were set based on the fluctuations of Q_*y*_ and CT energies along previous molecular dynamics simulations.^17^ The exciton domains were identified in this disordered picture by analyzing the participation ratio matrix (as done in our previous work.^45^)

The two model Hamiltonians were used to simulate absorption spectra within a non-Markov, secular approximation of the second-order cumulant expansion (see Section S3).^67^ We computed the homogeneous line shape using the same spectral density for all Chls, which was calculated with the vertical gradient method and previously employed by Saraceno et al.^34^ For CT states, the spectral density computed by Slama et al.^17^ was employed. The final absorption spectrum was obtained as average over the ensemble of 500 spectra calculated from the Hamiltonian realizations.

### 4.2 Kinetic Model

The rate of transfer among all Chls in the supercomplex was calculated according to the combined Redfield-Förster theory. The pigments are divided into domains that share strong exciton couplings; the intradomain dynamics transfers is treated with Redfield theory, while interdomain transfers are described with generalized Förster theory^40^ (see Section S4 for the equations used to compute the rates). The ground state was added to the matrix of the rates and connected to the lowest exciton of the RC domain with a fixed microscopic rate of 2 ps^–1^. This value results in an effective 1 ps^–1^ rate from the RC domain to the trap, which is consistent with what was estimated spectroscopically at room temperature^50^ (see Sections S4.1and S4.3). The energy transfer dynamics was calculated by solving the kinetic equation Ṗ(t)=KP(t) , where **P**(*t*) is the population vector at time *t* and **K** is the Redfield-Förster rate matrix expressed in the exciton basis; namely, *k*_*ab*_ is the rate from *b* to *a*, and *k*_*aa*_ = – *∑*_*b*_ *k*_*ba*_. The EET simulations were carried out using the python pyQME package.^68^

## Supplementary Material



## Data Availability

Supplementary data and pyQME code version employed in this work available at Zenodo: 10.5281/zenodo.20412101.

## Notes

Details on structure preparation; details on the exciton structure; simulation of absorption spectra; kinetic model; global analysis of populations; temperature dependence of the trapping time and light-harvesting efficiency and Supplementary Figure S3–S14 (PDF)
