# Supplementary material for: Energy Transfer Pathways in Plant Photosystem I from First-Principles Modeling
Source: JACS Au. 2026 Aug 11;6(8):4513–23. doi: 10.1021/jacsau.6c00568 (PMC13508078; doi:10.1021/jacsau.6c00568)
Supplement: Supplementary file 1 [file au6c00568_si_001.pdf]

Supporting Information: Energy transfer pathways in plant Photosystem I from first-principles modeling

Elena Betti*[1] and Lorenzo Cupellini*[1]

[1]Dipartimento di Chimica e Chimica Industriale, Università di Pisa, via G. Moruzzi 13, 56124 Pisa, Italy

*Corresponding Author, e-mail: elena.betti@phd.unipi.it; lorenzo.cupellini@unipi.it

S1 Details on structure preparation

The PSI-LHCI structure was downloaded from the PDB server (PDB ID: 5L8R)[S1]. Dodecyl-β-D-maltoside (LMT) and phylloquinone (PQN) residues were removed, as well as the SF_4 cluster. Missing waters, in particular those that coordinate Chls in the LHCI antenna, where added in analogy to available structures of LHCII[S2] and green algae[S3]. Chlorophyll and lipid tails not resolved in the map where reconstructed using the AMBER software *tleap* and their conformation fixed with a Monte Carlo procedure which minimizes clashes with existing residues. All residues were left in their standard protonation, except for histidines coordinating chlorophylls, whose δ- or ε- protonation was chosen so as to allow coordination. The ff14SB forcefield was used to describe the protein. Parameters for the Chls were taken from the literature.[S4] Lipid parameters were adapted from the lipid21 Amber forcefield.[S5] Carotenoids were modeled using parameters developed in previous works.[S6] The refinement then followed the protocol explained in Betti *et al.*[S7]

S2 Details on the exciton structure

Hamiltonians. The *noCT* exciton Hamiltonian of the PSI-LHCI aggregate is modeled as:

$$\hat{\mathcal{H}}_{noCT} = \sum_i \epsilon_i |i\rangle \langle i| + \sum_{i\neq j} V_{ij} |i\rangle \langle j| \tag{S1}$$

where ϵ_i is the vertical excitation energy of pigment i, denoted as *site energy*, and V_{ij} is the *excitonic coupling* between pigments i and j. Each state $|i\rangle$ is a local excitation on pigment *i*. Diagonalization of $\hat{\mathcal{H}}_{noCT}$ yields the exciton states $|a\rangle = \sum_i c_{ia} |i\rangle$, where c_{ia} represents the contribution of site i to exciton a with vertical energy E_a.

To include the red forms in the modeling, we extended the Hamiltonian above with CT states. We define $\hat{\mathcal{H}}_{CT}$ as:

$$\hat{\mathcal{H}}_{CT} = \hat{\mathcal{H}}_{noCT} + \sum_{k=1}^{4} \epsilon_k |k\rangle \langle k| + \sum_{ik} V_{ik}(|i\rangle \langle k| + |k\rangle \langle i|) \tag{S2}$$

where *k* runs over the 4 CT states, $|a603^+a609^-\rangle$ and $|a603^-a609^+\rangle$ for Lhca3/4, and V_{ik} are the couplings between the CT and LE states. These latter are nonzero only for those *i* states participating to the CT state

($|a603^*\rangle$ and $|a609^*\rangle$ in Lhca3/4). The CT energies ϵ_k and CT/LE couplings V_{ik} are taken from the work by Slama *et al.*[S8]. Therein, a diabatization precedure was employed to extract from the adiabatic excited states of the supermolecule formed by the a603/a609 dimer the localized states, with associated CT energies and CT-LE couplings. The calculation was repeated along many frames of MD simulations, resulting in different CT mixing. In order to build our *CT* model, we averaged only energies and couplings from the dimer configurations which resulted in a low-energy absorption band.

In Sláma *et al.,*[S8] the Tamm-Dancoff approximation (TDA) was employed, together with the ωB97X-D functional, to calculate the energies of the a603/a609 dimer. Here, instead, we employed full TD-DFT with the M06-2X functional to obtain the site energies. In order to preserve the CT-LE energy gap found by Sláma *et al.*, we shifted the CT energies by the difference between the mean of the site energies of a609/a603 in the TDA framework and in the TDDFT framework. The final values for ϵ_k and V_{ik} are given in Table S1.

**Lhca3**	**a603***	**a609***	**a603$^-$a609$^+$**	**a603$^+$a609$^-$**
**a603***	16810	270	603	-895
**a609***	270	16724	-897	662
**a603$^-$a609$^+$**	603	-897	21915	-19
**a603$^+$a609$^-$**	-895	662	-19	19346
**Lhca4**	**a603***	**a609***	**a603$^-$a609$^+$**	**a603$^+$a609$^-$**
**a603***	16737	270	603	-895
**a609***	270	16651	-897	662
**a603$^-$a609$^+$**	603	-897	21842	-19
**a603$^+$a609$^-$**	-895	662	-19	19273

Table S1: LE/CT energies and couplings of the a603-a609 dimer in Lhca3 and Lhca4.

Static disorder. In order to include static disorder in the model, we generated two separate ensembles of 500 Hamiltonians $\{\hat{\mathcal{H}}_{noCT}\}$, $\{\hat{\mathcal{H}}_{CT}\}$. We obtain them by sampling each site energy from a gaussian distribution centered on the computed site energy and with standard deviation $\sigma_{LE} = 100$ cm^{-1} (a typical fluctuation of site energies calculated on molecular dynamics simulations[S9]). For CT states, we use the same strategy but setting $\sigma_{CT} = 600$ cm^{-1}, according to typical fluctuations observed in molecular dynamics.[S8]

Exciton scheme. The exciton structure within a disordered framework can be studied using the exciton state pigment distribution functions, which quantify the contribution of a pigment at each exciton energy. The distribution function of pigment i reads[S10]:

$$d_i(\omega) = \left\langle \sum_a |c_{ia}|^2 \ \delta(\omega - \omega_a) \right\rangle_{\text{dis}} \tag{S3}$$

where c_{ia} are the excitonic coefficients and $\omega_a = E_a/\hbar$ is the frequency associated to exciton a, while $\langle \cdot \rangle_{\text{dis}}$ denotes an average over the disordered ensemble of Hamiltonians. We calculated the distributions for all pigments and then summed them according to their protein assignments. We obtained distributions along the exciton energies for all chains (Fig. S1), that were then schematized with rectangular boxes cut approximately at the inflection point of each curve in Fig. 2B of the main text. The zero energy is chosen so as to match the lowest peak of the RC density.

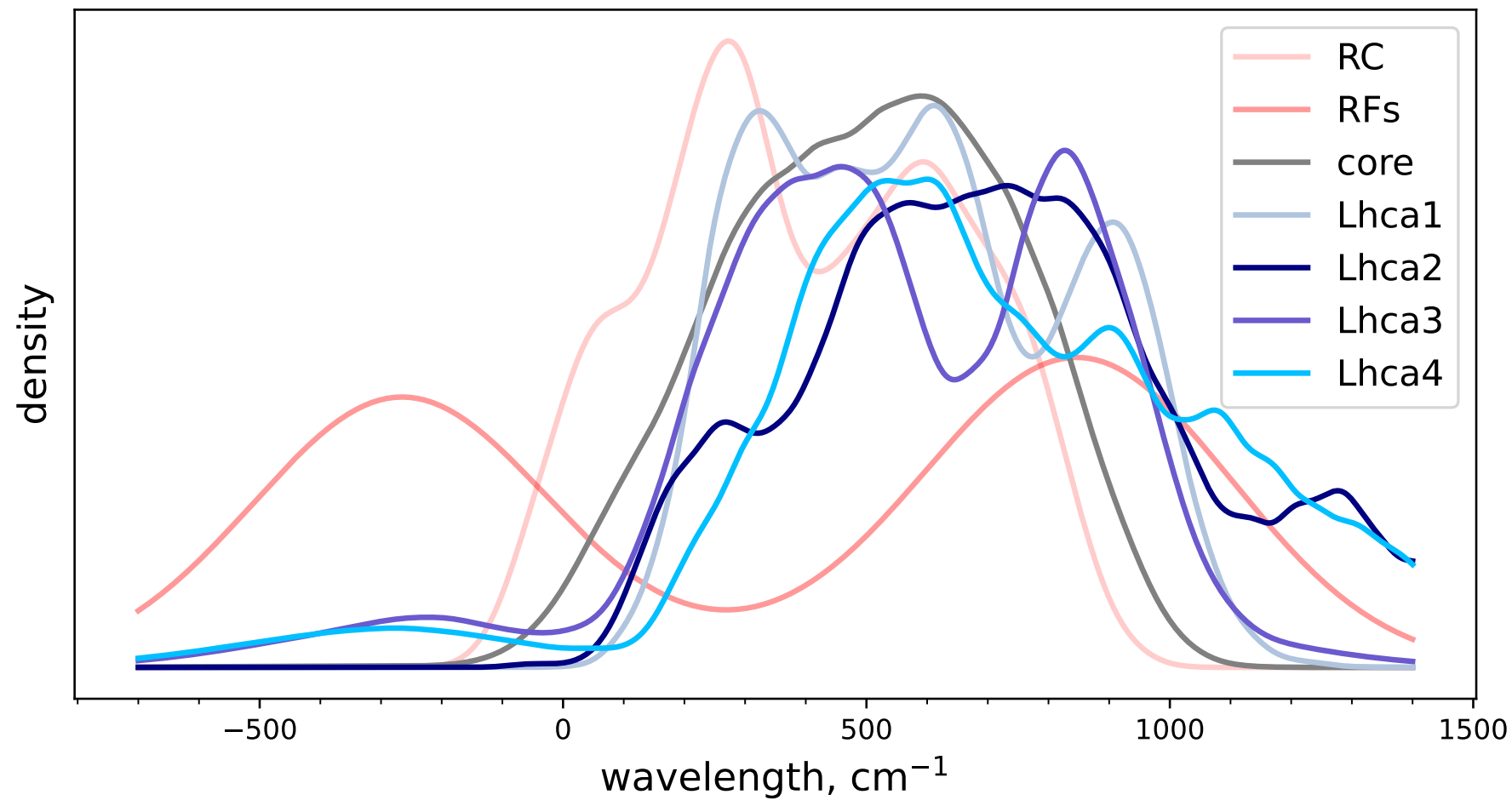

Figure S1: Exciton state distribution functions of the compartments underlying the boxes shown in Fig.2B of the main text.

S3 Simulation of absorption spectra

We simulated the absorption spectrum by using the cumulant expansion formalism and complex time-dependent Redfield (ctR) theory.[S11] In particular, we compute the absorption $A(\omega)$ of the excitonic aggregate as a sum over exciton states:

$$A(\omega) \propto \omega \sum_a |\mu_{0a}|^2 A_a(\omega) \tag{S4}$$

where $A_a(\omega)$ is the absorption of exciton a:

$$A_a(\omega) = \Re \int_0^{+\infty} dt \; e^{-i(\omega_a - \omega)t - g_a(t) - \xi_a(t)} \tag{S5}$$

Here $\omega_a = E_a/\hbar$ is the frequency associated to the exciton a, $g_a(t)$ is the lineshape function in the exciton basis, which can be calculated from the one in the site basis as:

$$g_a(t) = \sum_i |c_{ia}|^4 \, g_i(t) \tag{S6}$$

This latter is calculated from the spectral density $J_i(\omega)$

$$g_i(t) = -\int_0^{+\infty} d\omega \, \frac{J_i(\omega)}{\pi\omega^2} \left[\coth\left(\frac{\hbar\omega}{2k_B T}\right)(\cos(\omega t) - 1) - i(\sin(\omega t) - \omega t) \right]$$

Finally, $\xi_a(t)$ is the time-dependent term (non-Markov formulation) in the lineshape accounting for the dephasing of exciton a. We compute it according to:

$$\xi_a(t) = \sum_{b \neq a} \sum_i |c_{ia}|^2 |c_{ib}|^2 \int_0^t dt_2 \int_0^{t_2} dt_1 e^{i\omega_{ab} t_1} \mathcal{C}_i(t_1)$$

Here $\mathcal{C}_i(t)$ is the energy gap correlation function of site i and $\omega_{ab} = \frac{E_b - E_a}{\hbar}$ is the frequency associated to the energy gap between exciton a and b.

Here, the spectral density (SD) is assumed to be the same for all pigments (Chls a and b) for LE states. We use the average of the SDs calculated for CP29 by Saraceno *et al.* using a normal-mode analysis.[S12] For CT states, we employed the SD calculated by Slama *et al.* in Ref. S8.

The simulation of the absorption spectrum has been performed with the pyQME software[S13].

S4 Kinetic model

The dynamics is obtained upon solution of the Pauli master equation:

$$\dot{P}_a(t) = \sum_b k_{ab}\, P_b(t) - \sum_b k_{ba}\, P_a(t) \tag{S7}$$

or in matrix notation

$$\dot{\mathbf{P}}(t) = \mathbf{K}\mathbf{P}(t) \tag{S8}$$

where a and b are domain-localized exciton states (see below).

The transfer rates k_{ab} between exciton states were calculated according to Redfield-Förster theory. The Hamiltonian (here $\hat{\mathcal{H}}_{exc}$, which can either be $\hat{\mathcal{H}}_{noCT}$ or $\hat{\mathcal{H}}_{CT}$) is partitioned in domains of strong excitation:

$$\hat{\mathcal{H}}_{exc} = \hat{\mathcal{H}}_{exc,0} + \hat{V} \tag{S9}$$

where $\hat{\mathcal{H}}_{exc,0}$ contains the diagonal and all intra-domain couplings found in $\hat{\mathcal{H}}_{exc}$, while inter-domain couplings are treated as perturbations and moved to $\hat{V}$. The states a, b are eigenstates of $\hat{\mathcal{H}}_{exc,0}$.

Intra-domains transfer are described by Redfield theory:

$$k_{b\to a} = k_{ab} = \sum_i |c_{ia}|^2 |c_{ib}|^2 \left[1 + \coth\left(\frac{\beta\hbar\omega_{ab}}{2}\right)\right] J_i(\omega_{ab}) \tag{S10}$$

where c_{ia} and c_{ib} are excitonic coefficients, $J_i(\omega_{ab})$ is the spectral density of site i, $\beta = 1/k_B T$ is the inverse temperature and $\omega_{ab} = (E_b - E_a)/\hbar$, with E_a the energy of exciton a. Inter-domains transfers are described by Generalized Förster rates:

$$k_{b\to a} = k_{ab} = \frac{2|V_{ab}|^2}{\hbar^2} \Re \int_0^\infty e^{-i\omega_{ab} - 2i\lambda_b t - g_b(t) - \gamma_b^* t - g_a(t) - \gamma_a t} dt \tag{S11}$$

where

$$V_{ab} = \sum_{ij} c_{ia} c_{jb} V_{ij}$$

and V_{ij} are the elements left in the perturbation matrix V. The time integral quantifies the spectral overlap between the absorption of the acceptor state (see expression above for $A_a(\omega)$) and the fluorescence of the donor state. In the integral, λ_b is the reorganization energy of exciton b, and $g_b(t)$ is the excitonic lineshape function (eq. S6). The other quantities have the same meaning as above.

The reorganization energy is computed from the spectral density in the site basis as:

$$\lambda_i = \int_0^\infty d\omega\, \frac{J_i(\omega)}{\omega}$$

The (diagonal) reorganization energy of exciton a is

$$\lambda_a = \sum_i |c_{ia}|^4\, \lambda_i$$

The inclusion of the imaginary part of the dephasing term γ_a in eq. (S11) ensures that detailed balance,

$$\frac{k_{a\to b}}{k_{b\to a}} = e^{-\beta(\tilde{E}_b - \tilde{E}_a)},$$

is obtained according to the following effective energy

$$\tilde{E}_a = E_a - \lambda_a + \Im\gamma_a \tag{S12}$$

where $\Im\gamma_a$ is the imaginary part of the dephasing γ_a. The corrections at the right-hand side of eq (S12) respectively measure the diagonal and off-diagonal reorganization energy of the exciton.[S12]

The simulation of EET dynamics have been performed with the pyQME software[S13].

In order to simulate the trapping in the RC by charge separation, we added a trap state to the rate matrix. In each realization of the disorder, we connected this state to the lowest exciton state of the RC domain, with the same rate of 2 ps^{-1} (see below).

S4.1 Elementary trapping rate

To model trapping in the RC, we assume that only one exciton of the RC (the lowest in each disorder realization) is connected to the trap, while all the other excitons (including the other states in the RC) are given a zero rate to the trap. This is a model assumption needed to describe trapping, and does not reflect any calculation performed on the charge-separated state. Finally, we tune the elementary rate from the lowest RC exciton to the trap so as to provide an effective rate of about 1 ps from the RC domain to the trap, according to the 0.8 ps effective time for charge separation and to the 1–3 ps interval indicated by room temperature 2DES experiments.[S14]

More specifically, we note that an elementary rate from the lowest exciton state to the charge-separated state would result in a slower effective rate. We can estimate this effective rate from the RC to the trap assuming that the transfer occurs from an internally equilibrated RC domain. In mathematical terms[S7,S15]:

$$K_{\text{RC}\to\text{trap}} = \sum_{a\in\text{RC}}^{N=6} P_a^{eq}\, k_{a\to\text{trap}}$$

where P_a are the equilibrium populations within the RC compartment comprising the 6 exciton states, according to detailed balance in the Redfield Förster model, and $k_{a\to trap}$ is the microscopic rate from exciton a to the trap. Within our model, only the lowest of the 6 excitons in each realization is connected to the trap with a microscopic rate of $k_{a\to trap} = 2$ ps^{-1}. While the lowest exciton is always the most populated one, depending on the degeneration with other states in the RC, a significant part of the population can be found in the other states that are not connected to the trap, so that the effective rate $K_{RC\to trap}$ is reduced with respect to the microscopic one. In most of our disorder realizations, about half of the population is found in the lowest exciton, which results, on average, in a halved effective rate when compared to the microscopic one. An analysis of the sensitivity to the choice of the elementary trapping rate can be found below, in Section S4.3.

S4.2 Trapping times

The population dynamics within the *CT* and *noCT* models shown in Fig. 3B were fitted with a combination of exponential functions in order to extract the average trapping time. The parameters extracted from the fit are shown in Table S2. The average time τ_{avg} is the weighted average of the individual times, and is identical to the trapping time defined below in eq. (S13). With the multiexponential fitting, we further show that the

noCT model generates an almost perfect monoexponential decay, while the decay obtained with the *CT* model is markedly multiexponential.

	A_1	τ_1	A_2	τ_2	A_3	τ_3	τ_{avg}
noCT	1	33	-	-	-	-	33
CT	35	15	55	58	10	152	54

Table S2: Parameters from multiexponential fitting of CT and noCT population decay.

The trapping times shown in Table 1 of the main text are computed as mean first-passage times (MFPT) from the Redfield-Förster rates. They represent the expected time for an initial excitation to reach the trap state. They are equivalent to the τ_{avg} obtained from the multi-exponential fitting of populations.

Given an initial condition $\mathbf{P}^x(0)$, the MFPT can be computed according to:

$$\langle \tau \rangle^x = \sum_{a \neq \text{trap}} \int_0^\infty P_a^x(t)\, \mathrm{d}t = \sum_{a \neq \text{trap}} \tau_a^{r;x} \tag{S13}$$

where $P_a^x(t)$ is the population on state a at time t given the initial condition x, whereas $\tau_a^{r;x}$ is the residence time of the excitation on state a upon initial condition x. The sum of the time integrals is extended to all states except the trap. The residence time is obtained directly from the rate matrix K as:

$$\tau_a^{r;x} = -\sum_b [\tilde{K}^{-1}]_{ab}\, \tilde{P}_b(0) \tag{S14}$$

where $\tilde{K}^{-1}$ indicates the inverse of the matrix $\tilde{K}$, which is obtained from K upon removal of the line and column corresponding to the trap. Similarly, $\tilde{P}_b(0)$ is element *b* at time zero of the population vector $\tilde{\mathbf{P}}$, obtained by excluding the trap state from **P**.

S4.3 Sensitivity to the elementary trapping rate

In this section we assess the sensitivity of the kinetics to the choice of the elementary trapping rate. In particular, we report the trapping times upon homogeneous excitation obtained by setting the trapping rate equal to 1, 1.6 and 2 ps^{-1} (the latter being the one used for this work). These elementary rates result in *effective* trapping timescales from the RC to the trap of 2, 1.25 and 1 ps respectively, which roughly explore the interval indicated by 2DES experiments.[S14] In the table below, we show the trapping times upon homogeneous excitation for both CT and noCT models.

	1 ps^{-1}	1.6 ps^{-1}	2 ps^{-1}
noCT	47	37	33
CT	74	60	55

Table S3: Trapping times (ps) upon homogeneous excitation with different elementary trapping rates.

S4.4 Calculation of population fractions in RFs

The fraction of excitation visiting the RFs before reaching the trap can be calculated by considering the RF states as another trap, that is, by removing all rates out of the RF states. In practice, we set to zero all rates outgoing from the lowest exciton state of each RF domain. The fraction of excitation visiting the RFs is equal to the

population of the RF lowest exciton at time $t = \infty$. Given each initial condition, we calculate the population vector in the exciton basis at full convergence. In Table 1 we show the cumulative population of all RF states.

S4.5 Fluxes

We compute fluxes of population between all exciton states for each frame of the dynamics. The flux $\Phi^{x}_{a\to b}$ quantifies the net probability of transfer from a to b upon a given initial condition x, defined by the vector of populations $\mathbf{P}^{x}(0)$, and is calculated as:

$$\Phi^{x}_{a\to b} = k_{ba}\tau^{r,x}_{a} - k_{ab}\tau^{r,x}_{b} \tag{S15}$$

where k_{ab} are the elements of the rate matrix K and $\tau^{r,x}_{a}$ are the residence times defined above. $k_{IJ}\tau^{r,x}_{J}$ can be thought of as the average number of $J \to I$ hops. In order to perform disorder averages, we compute fluxes between domains Φ^{x}_{IJ} by summing all fluxes between states in I and J and then average them over the ensemble. The total flux out of domain J can be computed (dropping the superscript x for simplicity):

$$\phi^{\text{out}}_{J} = \sum_{I} \max(0, \Phi_{J\to I})$$

since positive fluxes signify net transfer *out* of the domain.

S5 Global Analysis of populations

To analyze our kinetic data, we perform a global analysis. That means, given a dataset of the form $P(t, S)$ where S is a species, we find the best-fit exponentials $\{e^{-t/\tau_i}\}$ and respective amplitudes $\{A_i\}$ that describe the dynamics of all species, as already done for CP29 in Ref. S12. The global fitting was performed using the skultrafast package[S16], which was developed to analyze datasets from time-resolved spectroscopies. As described in the main text, we consider as species 5 comparments, obtained by summing the respective pigments population dynamics: Core, LHCI blue, RFs in Lhca3, RFs in Lhca4, RC. We repeat the analysis for three initial conditions: homogeneous, Core, LHCI blue excitation.

For the last two, we analyze the obtained amplitudes and associated timescales to estimate the effective times and pathway yields for all transfers in the schemes (Fig. 5B,D). The branching of the excitation (percent) is estimated from individual f_{RF-4} and f_{RF-3}, whose sum is shown in Table 1 for different excitation conditions. The timescale of the transfer from compartment A (initially excited) to compartment B (directly connected to A) is estimated by doing a weighted average of all τ_i for which A has negative amplitude and B has positive amplitude. While this neglects transfers between compartments connected within themselves, the estimate reasonably holds for the first ps of the dynamics. The decay of all compartments is similarly estimated as weighted average of all τ_i for which they feature negative amplitude. In this case, it is not possible to assign the decay to a particular pathway (because of the model's coarse-grained nature), so we simply assign an effective decay time of the excitation from that compartment.

S6 Temperature dependence of the trapping time and light-harvesting efficiency

All the analyses on the EET dynamics presented in this work were carried out at room temperature. As we demonstrate in the main text, independently of the excitation condition, a non-negligible part of the excita-

tion visits the red forms before trapping in the RC, meaning that equilibration of the network then requires thermally-activated uphill transfers. Temperature is therefore expected to have a large impact on the time and yield of trapping in the RC. To assess this, we compute rates and mean first-passage time (MFPT) at different temperatures in the range 77 K – 300 K and compare the resulting trapping times upon different initial conditions. Since at low temperatures the migration to the RC becomes comparable in terms of timescales to the natural fluorescence lifetime of Chls (≈ 2 ns) we now add this rate to the ground state from all excitons (this latter being the reason for slightly shorter times at 300K with respect to those shown in the main). The resulting MFPTs are reported here below:

Initial excitation	4 K	77 K	150 K	250 K	300 K
Homogeneous	1419	590	232	65	52
LHCI blue	1821	1217	469	105	76
Core	1215	265	110	45	40

Table S4: Trapping times (in ps) within the *CT* model at different temperatures upon specified initial conditions.

As expected, all the trapping time increase when decreasing temperature, as uphill transfers are progressively slowed down. At 77K, Akhtar *et al.*[S17] reported a typical timescale for equilibration in PSI-LHCI of 500 ps, which well compares to our 590 ps. At 4 K, a large part of the excitation decays with timescale close to the natural fluorescence decay. To quantify this observation, we estimate the photochemical yield of PSI at the different temperatures by computing the partition of the initial population between the RC trap and the added GS. Results are plotted in Fig.S2: the photochemical yield of the RC drops below 90% already below 250 K and is estimated to be only ≈30% at 4 K.

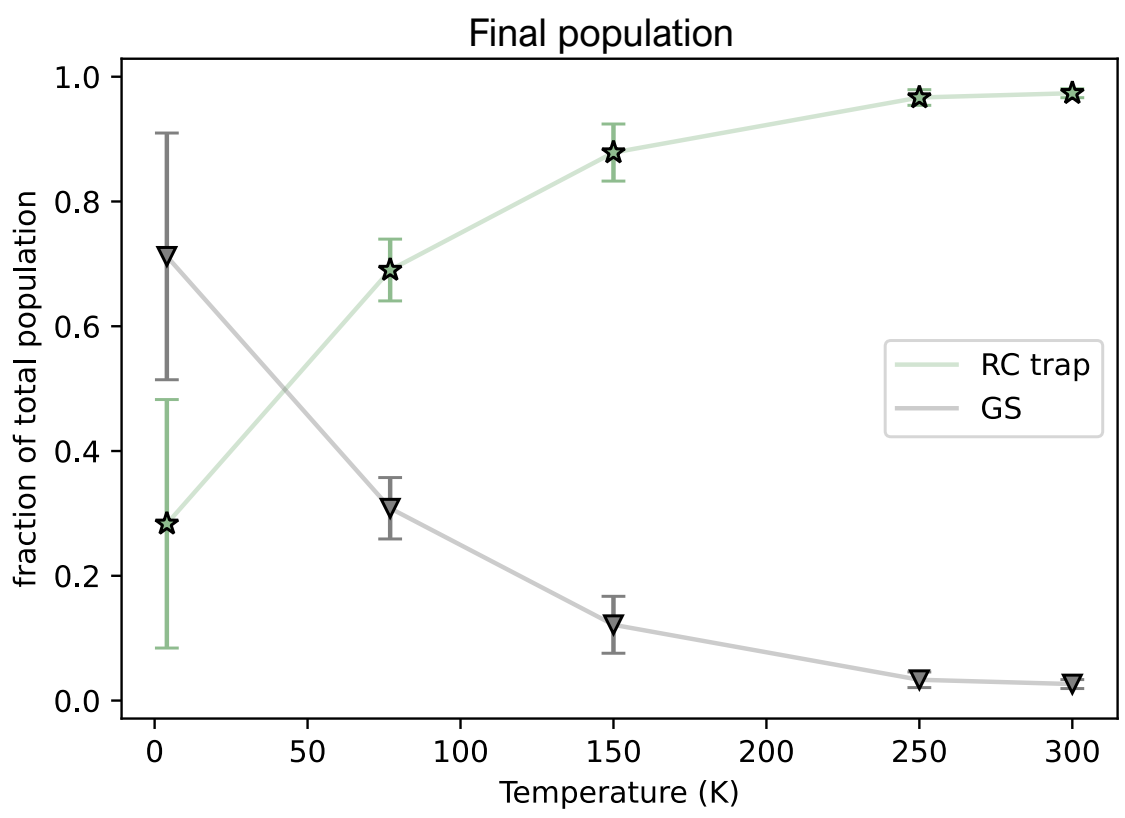

Figure S2: Partition of the initial population between the ground state (GS) and the reaction center trap (RC trap) as a function of temperature. Error bars indicate one standard deviation across the disorder realizations.

Indeed, at cryogenic temperatures the uphill transfer is essentially suppressed and the major part of the excitation is trapped in RFs as well as other local minima of the free energy in the EET network and fluoresce before reaching the RC. The trapping of excitation in the RFs at low temperature is also demonstrated by the 77 K static fluorescence of PSI, whose major peak is located above 730 nm, and blueshifts in mutants where the RFs are suppressed.[S18]

The errorbar in Fig.S2, indicating one standard deviation of the distribution along static disorder, also shows a marked trend with the temperature: the same slight changes in the energetic landscape result at low temperature in a large variability of the final conditions, since only few possibly irreversible pathways (compatible with

the available thermal energy) can be explored in each realization of disorder. Instead, at 300 K the thermal energy allows to explore the network more dynamically resulting in similar final ratios.

On a side, we stress that the extrapolation of our model to low temperature is expected to cause a larger uncertainty in the estimation of timescales, since the CT energies and couplings were calculated on a room-temperature molecular dynamics trajectory. Given the sharp sensitivity of CT states to the intermolecular geometry, the energy of CT states may be less accurate when extrapolating at low temperature.

Supplementary Figures

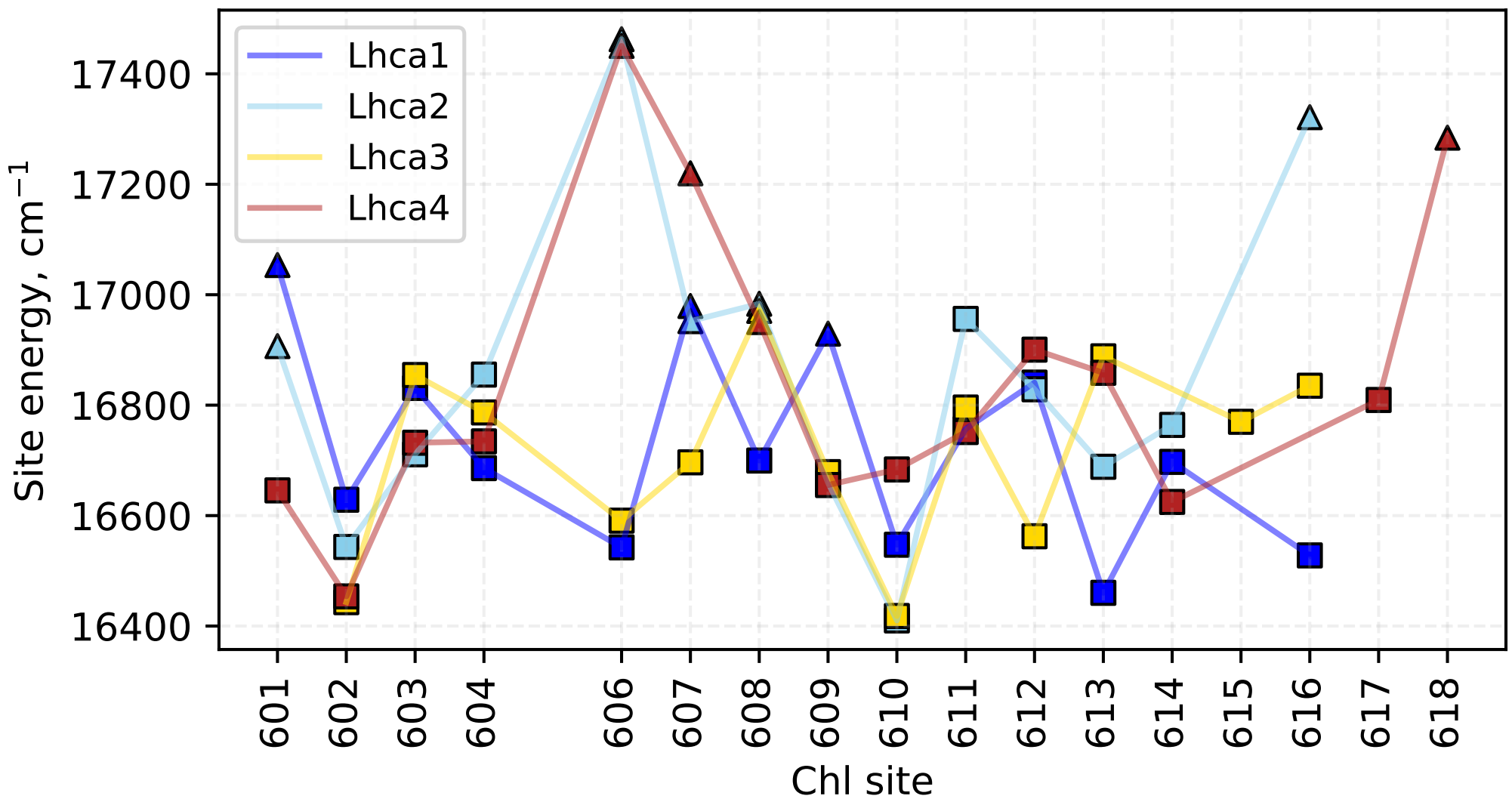

Figure S3: QM/MMPol site energies of Chls in the four Lhca antennas. Squares identify Chls a and triangles Chls b. The four chains are distinguished by color.

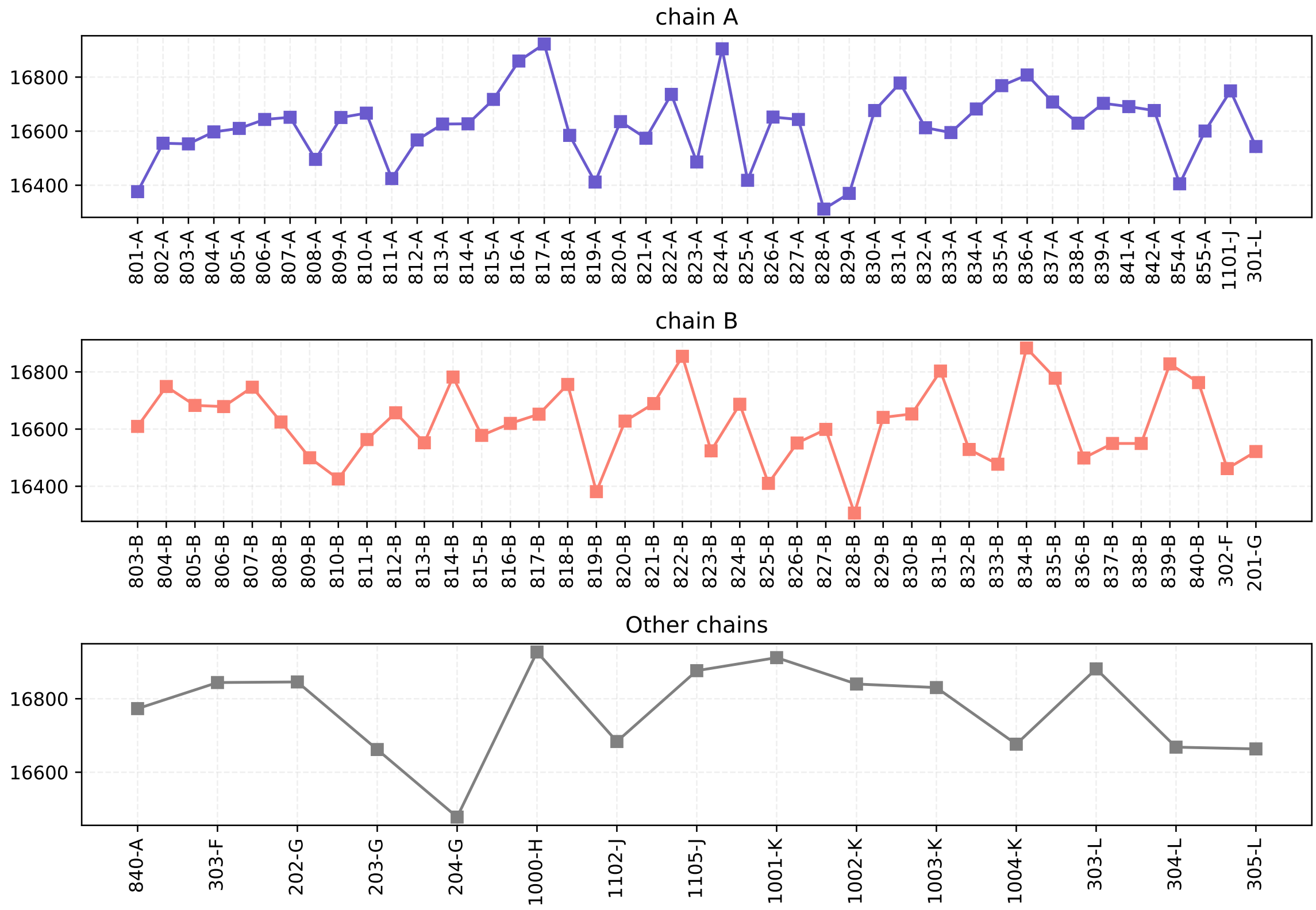

Figure S4: QM/MMPol site energies of Chls in the core complex.

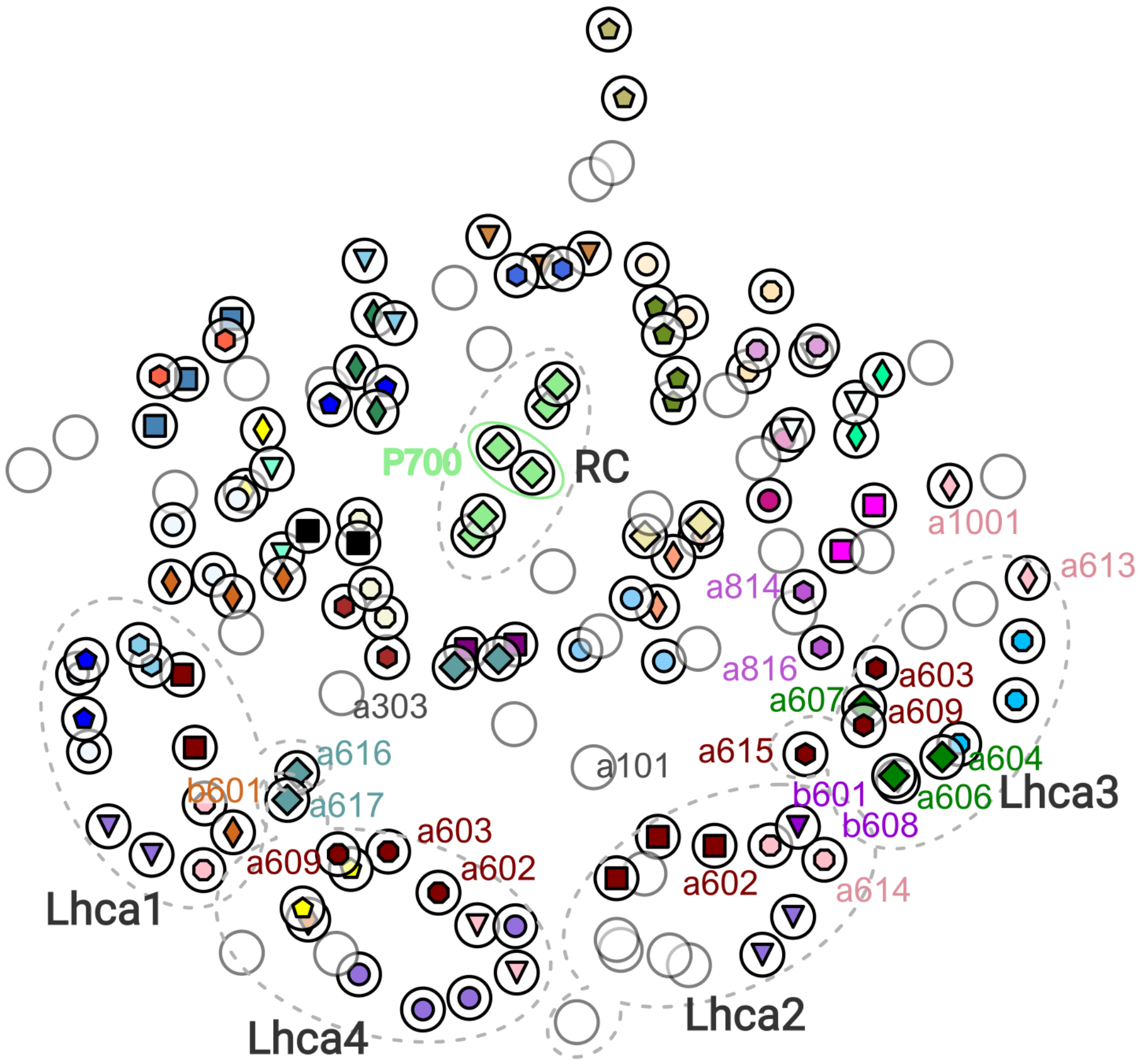

Figure S5: Graphical depiction of the exciton domains as found in this work. Each circle represents a Chl; empty circles stand for single-pigment domains, while pigments sharing the same symbol and the same color are part of the same domain. In Lhcas, domains including Chls a603/a609 are shown in dark red, Chl a610/a611/a612 in indigo, Chls a613/a614 in pink. Chls mentioned in the text are explicitly labeled.

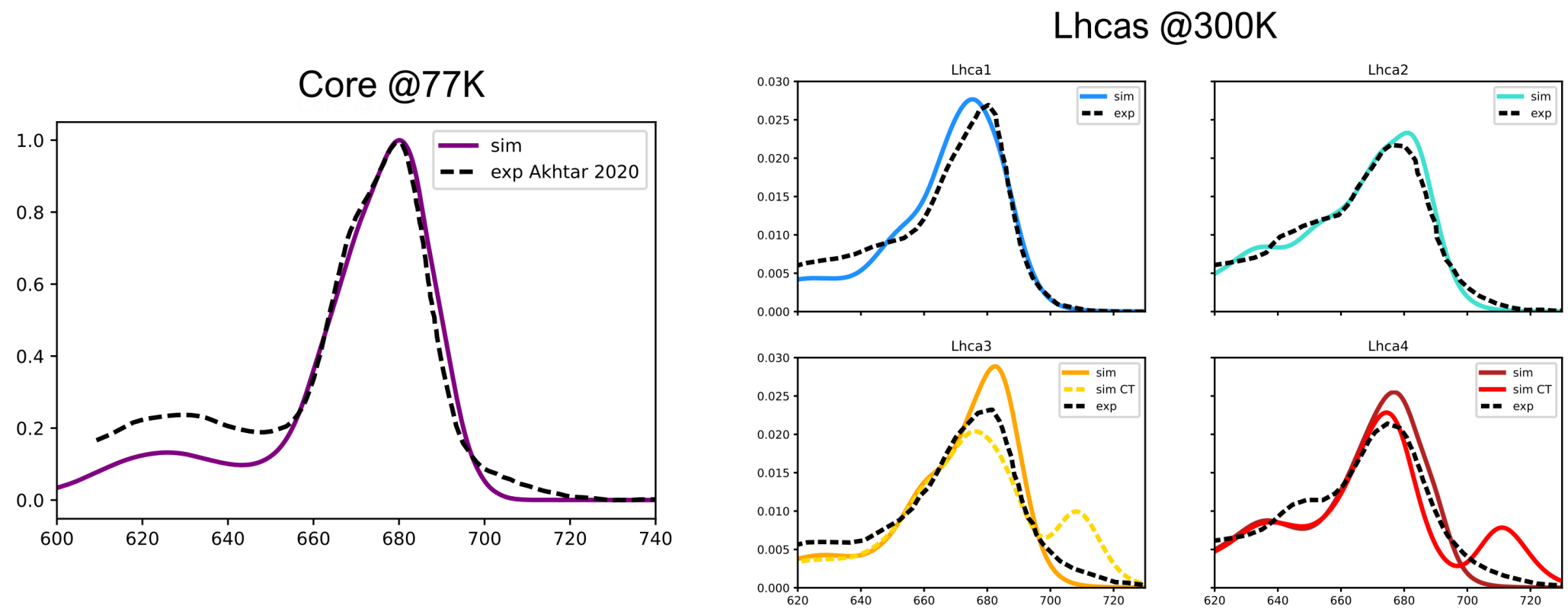

Figure S6: Absorption spectra of selected complexes in PSI-LHCI, obtained extracting from the full Hamiltonian the subset of pigments assigned to the protein chain. The good agreement of single complexes' spectra with experiment validates the reliability of the model. Left: low temperature (77 K) absorption of the core complex, normalized to the maximum. Experimental data digitalized from Akhtar *et al.*[S17] Right: room temperature absorption of individual LHCI antennas, normalized to the total area of the Q_y band (620-730 nm). Experimental data digitalized from Croce *et al.*[S19] (Lhca1 and Lhca4) and Castelletti *et al.*[S20] (Lhca2 and Lhca3).

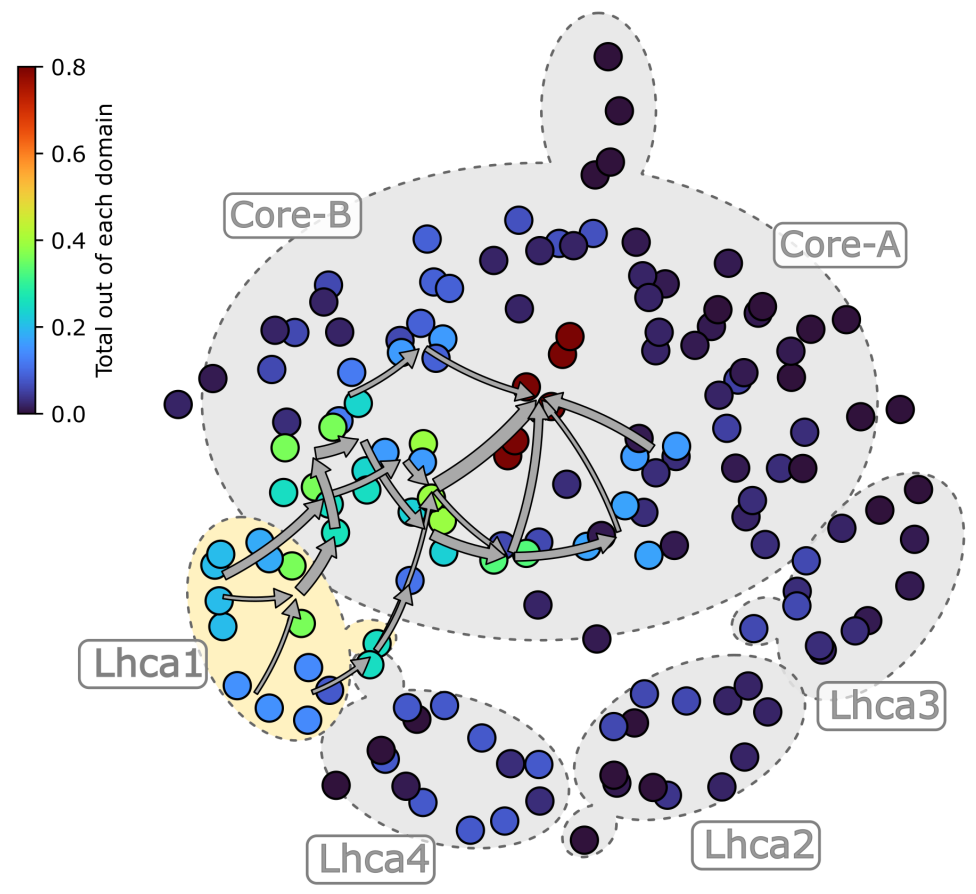

Figure S7: Fluxes with starting excitation on Lhca1 (left) and Lhca2 (right). Arrows thickness is proportional to the flux (above a threshold of 7 and 5% respectively).

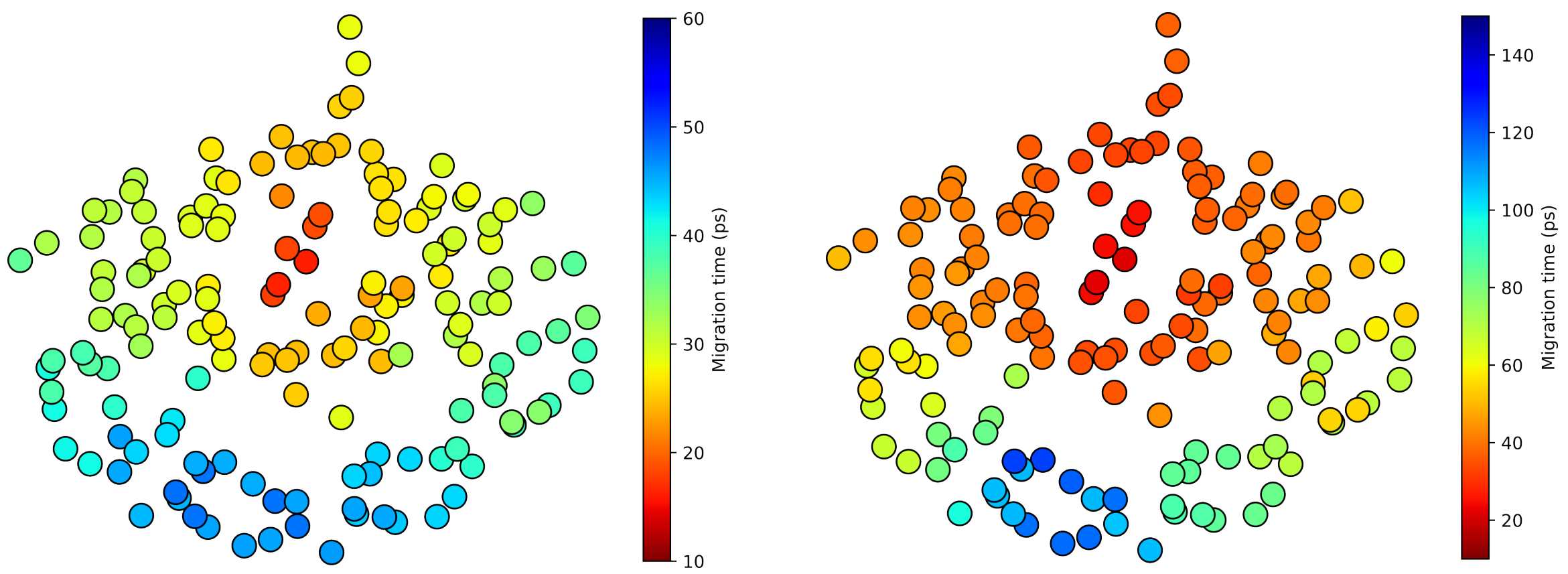

Figure S8: Pigments colored by the disorder-averaged migration time predicted upon their initial excitation, according to eq.S13. Left: *no CT* model; right: *CT* model. Note the different limit set for the colorbar.

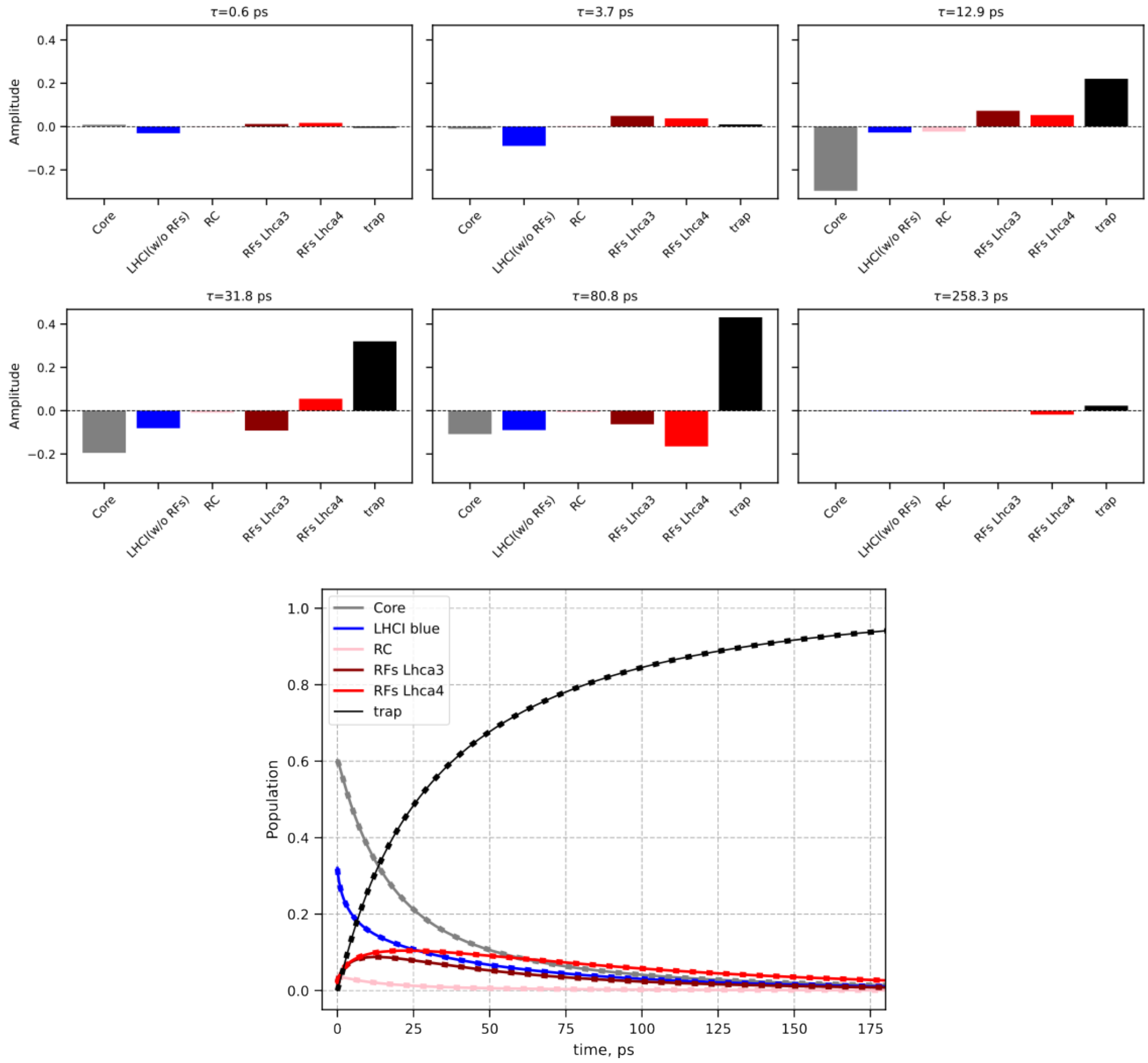

Figure S9: Global analysis of population dynamics after homogeneous excitation. Top: amplitudes associated to the fitted times for each compartment. Bottom: computed dynamics upon homogeneous excitation, summed for pigments within each compartment (solid) and fitted with the global analysis (dotted).

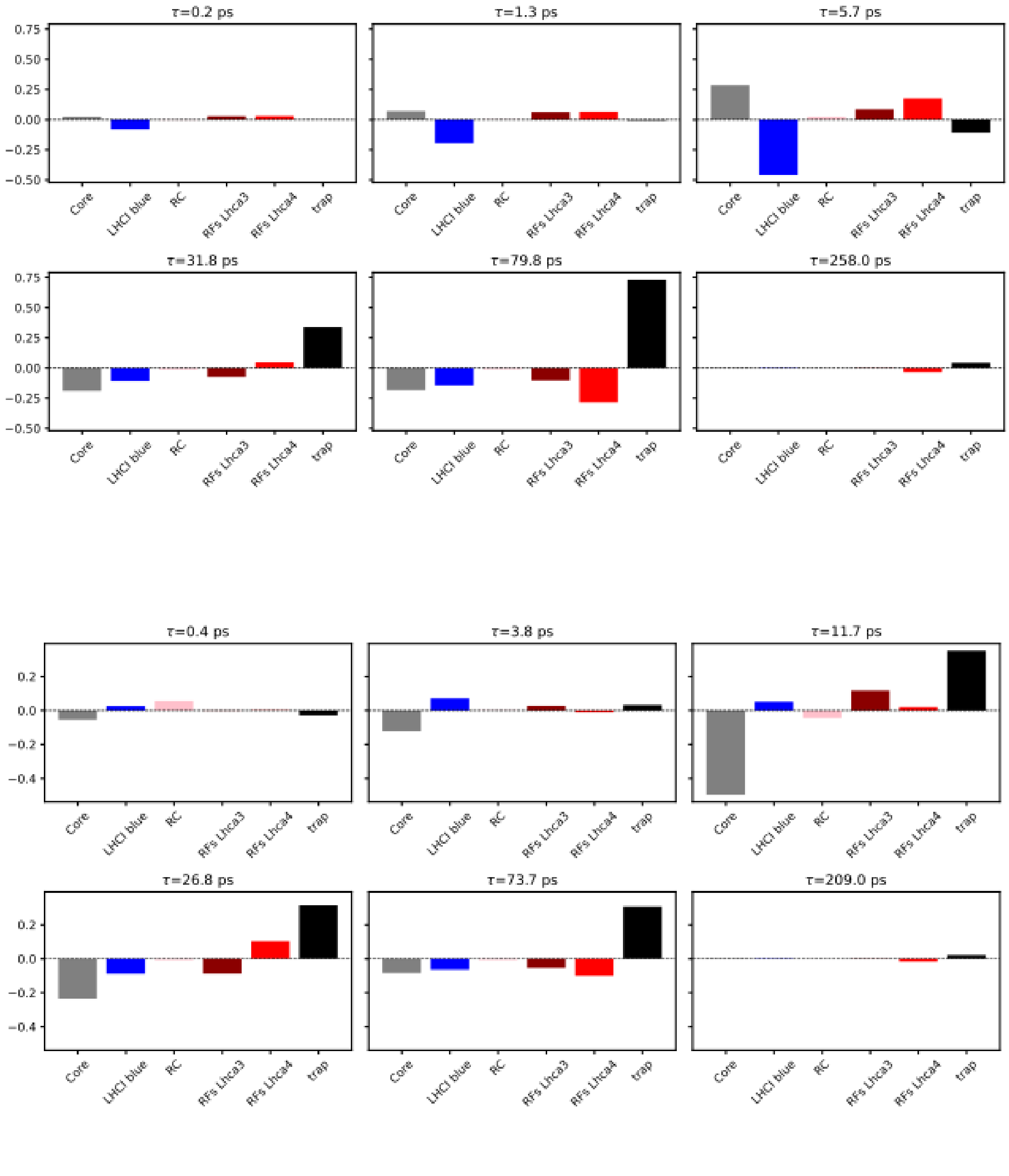

Figure S10: Amplitudes fitted for global analysis of population dynamics after excitation of the LHCI antenna (top) and core (bottom).

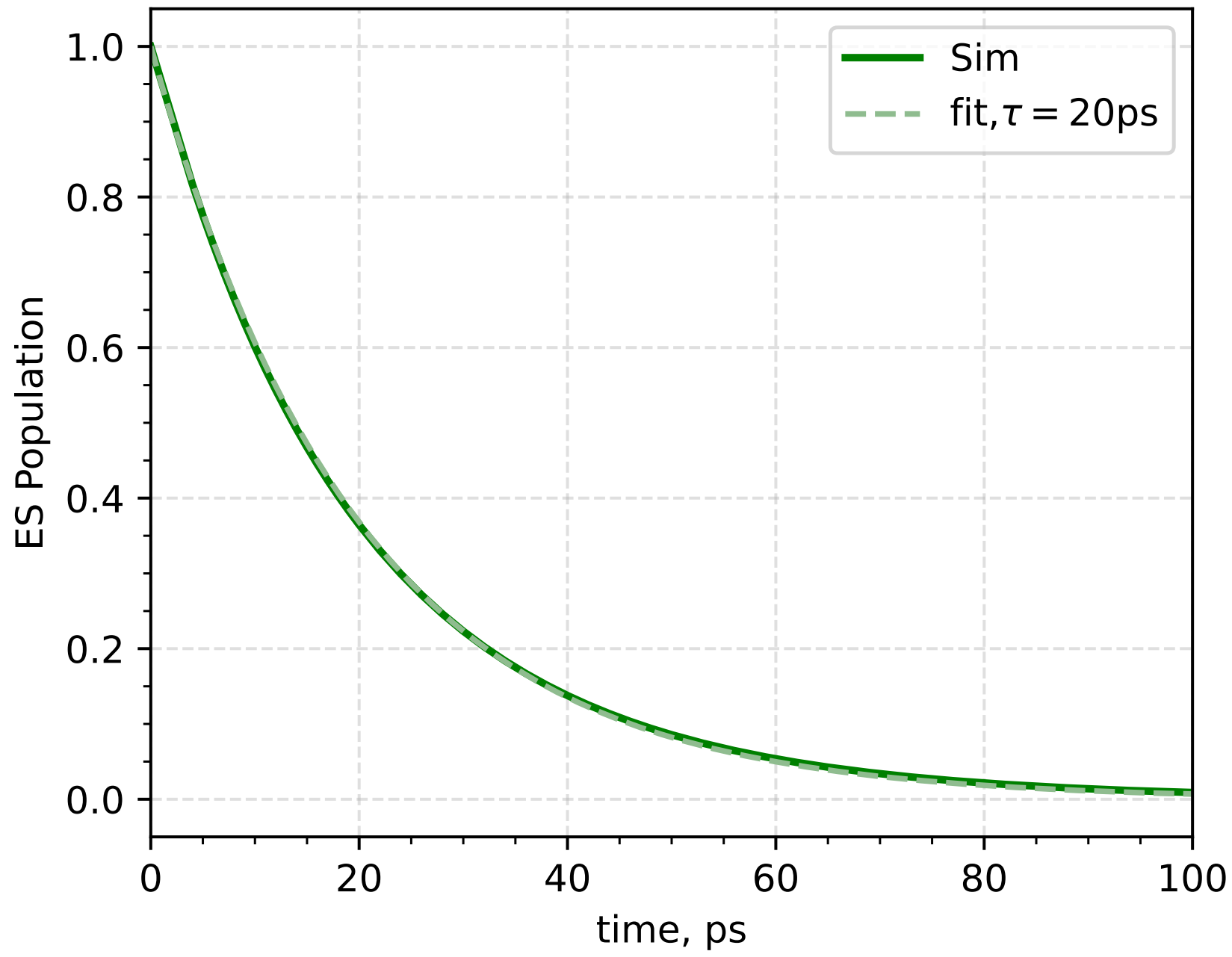

Figure S11: Population decay of the core model after homogeneous excitation. The core model was obtained by closing all EET channels out of the core. A single exponential $e^{-t/\tau}$ with $\tau = 20$ ps was used to fit the data.

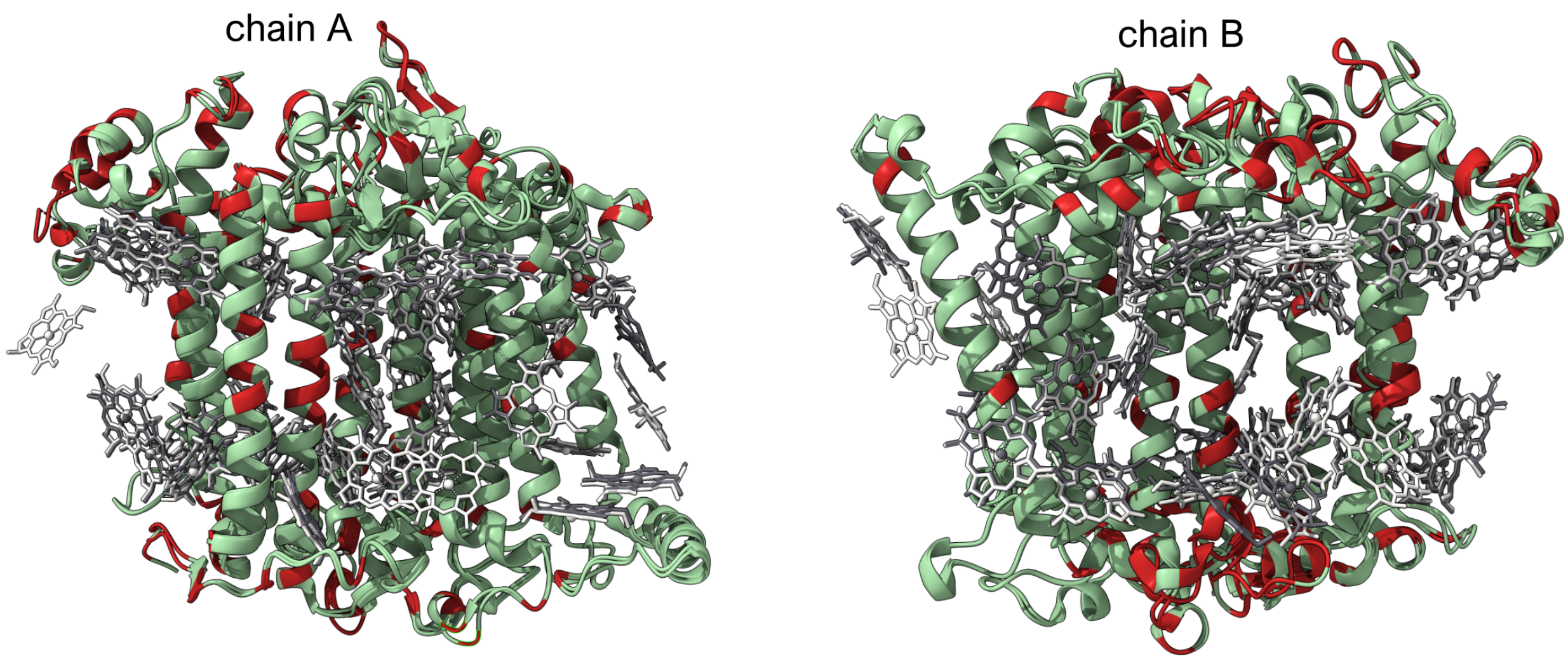

Figure S12: Structural comparison between cyanobacterial (PDB:1JB0[S21]) and plant (PDB:5L8R[S1]) chains A and B. Conserved residues are colored in green, mutated ones in red. Chls rings from both structures are also shown in white and grey for cyanobacterium and plant, respectively.

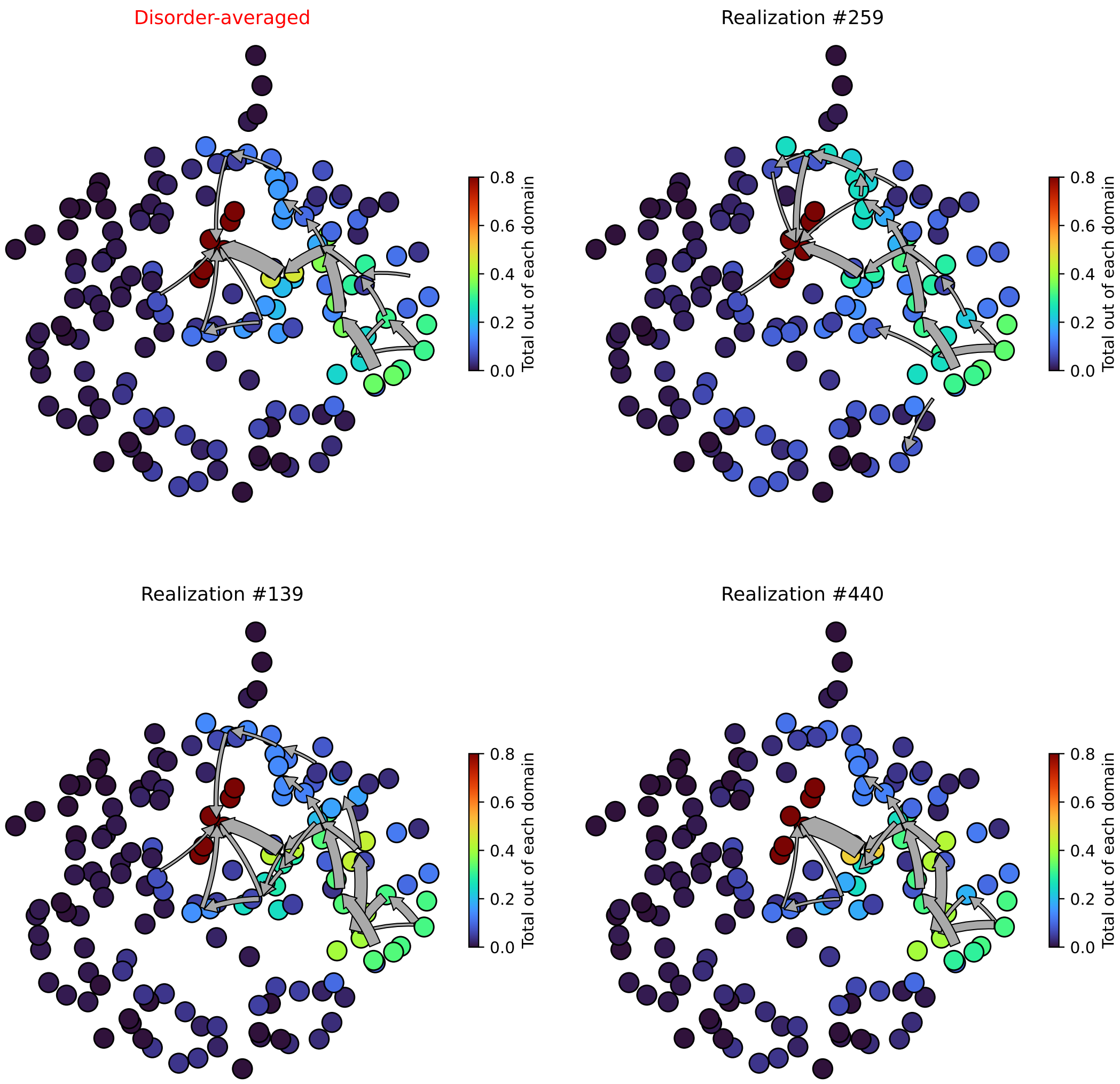

Figure S13: Population fluxes upon Lhca3 excitation (homogeneous except for red sites). Arrows indicate fluxes above 7%. The first panel shows the disorder-averaged fluxes, the other three show the fluxes as computed in individual realizations, chosen as the ones giving the largest deviation from the average one.

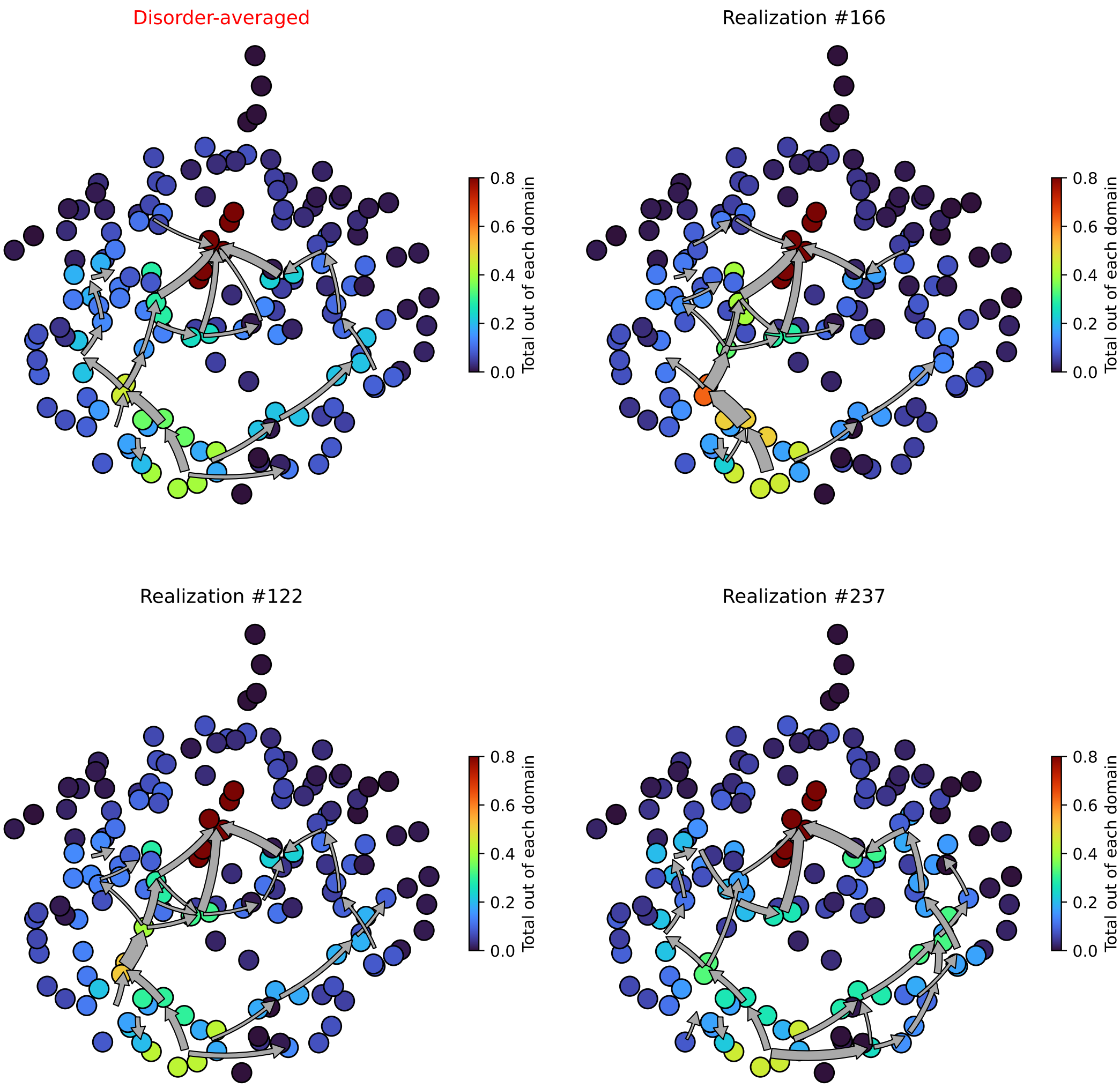

Figure S14: Population fluxes upon Lhca4 excitation (homogeneous except for red sites). Arrows indicate fluxes above 7%. The first panel shows the disorder-averaged fluxes, the other three show the fluxes as computed in individual realizations, chosen as the ones giving the largest deviation from the average one.

References

[S1] Mazor, Y., Borovikova, A., Caspy, I. & Nelson, N. Structure of the plant photosystem i supercomplex at 2.6 å resolution. *Nat. Plants* **3**, 17014 (2017). URL http://dx.doi.org/10.1038/nplants.2017.14.

[S2] Liu, Z. *et al.* Crystal structure of spinach major light-harvesting complex at 2.72 å resolution. *Nature* **428**, 287–292 (2004). URL http://dx.doi.org/10.1038/nature02373.

[S3] Naschberger, A. *et al.* Algal photosystem i dimer and high-resolution model of psi-plastocyanin complex. *Nat. Plants* **8**, 1191–1201 (2022). URL http://dx.doi.org/10.1038/s41477-022-01253-4.

[S4] Reiss, K., Morzan, U. N., Grigas, A. T. & Batista, V. S. Water network dynamics next to the oxygen-evolving complex of Photosystem II. *Inorganics* **7**, 39 (2019).

[S5] Dickson, C. J., Walker, R. C. & Gould, I. R. Lipid21: Complex lipid membrane simulations with amber. *J. Chem. Theory Comput.* **18**, 1726–1736 (2022).

[S6] Prandi, I. G., Viani, L., Andreussi, O. & Mennucci, B. Combining classical molecular dynamics and quantum mechanical methods for the description of electronic excitations: The case of carotenoids. *J. Comput. Chem.* **37**, 981–991 (2016).

[S7] Betti, E., Cupellini, L. & Mennucci, B. Quantum chemical modeling of energy flow and photoprotection in photosystem ii. *Cell Rep. Phys. Sci.* **7**, 103099 (2026). URL http://dx.doi.org/10.1016/j.xcrp.2026.103099.

[S8] Sláma, V. *et al.* Origin of low-lying red states in the lhca4 light-harvesting complex of photosystem i. *J. Phys. Chem. Lett.* **14**, 8345–8352 (2023). URL http://dx.doi.org/10.1021/acs.jpclett.3c02091.

[S9] Betti, E., Saraceno, P., Cignoni, E., Cupellini, L. & Mennucci, B. Insights into energy transfer in light-harvesting complex II through machine-learning assisted simulations. *J. Phys. Chem. B* **128**, 5188–5200 (2024).

[S10] Raszewski, G., Saenger, W. & Renger, T. Theory of optical spectra of Photosystem II reaction centers: Location of the triplet state and the identity of the primary electron donor. *Biophys. J.* **88**, 986–998 (2005).

[S11] Gelzinis, A., Abramavicius, D. & Valkunas, L. Absorption lineshapes of molecular aggregates revisited. *J. Chem. Phys.* **142**, 154107 (2015).

[S12] Saraceno, P., Sláma, V. & Cupellini, L. First-principles simulation of excitation energy transfer and transient absorption spectroscopy in the cp29 light-harvesting complex. *J. Chem. Phys.* **159**, 184112 (2023).

[S13] Saraceno, P. & Cupellini, L. pyQME: Package for open quantum system dynamics and spectroscopies simulations of excitonic systems. https://github.com/Molecolab-Pisa/pyQME (2023).

[S14] Akhtar, P. *et al.* Two-dimensional electronic spectroscopy of a minimal photosystem i complex reveals the rate of primary charge separation. *J. Am. Chem. Soc.* **143**, 14601–14612 (2021). URL http://dx.doi.org/10.1021/jacs.1c05010.

[S15] Bennett, D. I. G., Amarnath, K. & Fleming, G. R. A structure-based model of energy transfer reveals the principles of light harvesting in Photosystem II supercomplexes. *J. Am. Chem. Soc.* **135**, 9164–9173 (2013).

[S16] Stensitzki, T. skultrafast - a python package for time-resolved spectroscopy (2021). URL https://zenodo.org/record/5713589.

[S17] Akhtar, P. & Lambrev, P. H. On the spectral properties and excitation dynamics of long-wavelength chlorophylls in higher-plant photosystem i. *Biochim. Biophys. Acta, Bioenerg.* **1861**, 148274 (2020). URL http://dx.doi.org/10.1016/j.bbabio.2020.148274.

[S18] Capaldi, S. *et al.* Structural determinants for red-shifted absorption in higher-plants photosystem i. *New Phytol.* **248**, 2331–2346 (2025). URL http://dx.doi.org/10.1111/nph.70562.

[S19] Croce, R., Morosinotto, T., Castelletti, S., Breton, J. & Bassi, R. The lhca antenna complexes of higher plants photosystem i. *Biochim. Biophys. Acta, Bioenerg.* **1556**, 29–40 (2002). URL http://dx.doi.org/10.1016/S0005-2728(02)00304-3.

[S20] Castelletti, S. *et al.* Recombinant lhca2 and lhca3 subunits of the photosystem i antenna system. *Biochemistry* **42**, 4226–4234 (2003). URL http://dx.doi.org/10.1021/bi027398r.

[S21] Jordan, P. *et al.* Three-dimensional structure of cyanobacterial photosystem i at 2.5 å resolution. *Nature* **411**, 909–917 (2001). URL http://dx.doi.org/10.1038/35082000.
